# Adaptive control of magnetic levitation system based on fuzzy inversion

**DOI:** 10.1038/s41598-024-76067-9

**Published:** 2024-10-22

**Authors:** Marcin Jastrzębski, Jacek Kabziński

**Affiliations:** Institute of Automatic Control, Lodz University of Technology, 18 Stefanowskiego St, Lodz, 90-537 Poland

**Keywords:** Magnetic levitation system, Adaptive control, Nonlinear control, Fuzzy model inversion, Electrical and electronic engineering, Applied mathematics

## Abstract

A novel adaptive tracking controller for magnetic levitation systems (MLS) is developed. The controller is based on a special adaptive control scheme incorporating fuzzy model of electromagnetic acceleration enabling fast and accurate fuzzy inversion. The controller ensures accurate tracking of any smooth desired position signal, despite unknown MLS parameters. The closed-loop system stability, in the sense of uniform ultimate boundedness (UUB) of error trajectories, is proved using Lyapunov approach. The closed-loop system performance is investigated during numerical experiments. Finally the proposed controller is verified by successful implementation on a DSP board controlling a typical magnetic levitation ball system. Performed tests and experiments demonstrate that the proposed control technique is robust against discretization and unknown MLS model parameters, provides high tracking accuracy, is easily implementable, and simple to tune.

## Introduction

Magnetic levitation is a modern technology that is a subject of intensive research. It is known for its numerous spectacular applications, and attracts wide public interest. By magnetic levitation system (MLS) we mean any device that uses a controlled magnetic field to suspend an object and move it in a specific direction. Contactless, frictionless, and noiseless motion caused by MLS has been successfully applied to numerous fields of advanced technology, such as:

- high-speed maglev trains^1^,
- magnetic bearings^2^,
- maglev wind turbines^3^,
- microrobotics^4^,
- vibration control^5^.
- contactless suspension in precise measurement systems or sensors such as gyroscopes^6^,

or even for artistic activity such as floating sculptures using magnetic levitation. On the other hand, a simple MLS is easy to design, manufacture and study at the engineering degree level, and many universities are equipped with such MLSs that can be studied by students.

Since any MLS is an open-loop unstable, nonlinear control system, finding a high-performance control algorithm for controlling the position of the moving part of MLS is an opened challenge. This demonstrates once again that even advanced technology is useless without proper control. The problem becomes even more challenging if we consider not only position stabilization but also tracking the desired position, which is what we do in this paper and which is more interesting for various applications.

Over the years, several approaches have been developed to control position in MLS. Several recent review papers^7–9^ indicate the main directions of recent development of control algorithms, unanimously classifying them into three groups: classical linear control, nonlinear control and intelligent control.

Classical methods are based on linearization of the system around the operating point and the use of linear controllers, mainly PID^10^ However, the linearity of the controller means that the control system properties will change with the operating point. Furthermore, a control system tuned to limit overshoot will be characterized by an increase in settling time^8^. For this reason, it is necessary to apply other controllers that can cope with complex, nonlinear dynamics and use more than just two (position and velocity) state variables. Feedback control using a full-order observer is presented in^11^. Another application of the observer is to use it for predictive control with a nonlinear model^12^. In addition to estimating state variables, it is also possible to observe disturbances, e.g. a variable mass, as it was done in^13^. It is also possible to implement LQR^14^ or LQG^15^ techniques to obtain less variability of state variables in the presence of disturbances or noise, with less control effort. Extended or feedback linearization offers several possibilities to take into account the nonlinear nature of MLS^16–18^. Although these different control techniques can achieve good control results when evaluated from different perspectives, there is still space for improvement in the control performance of MLSs.

Recently, it was observed that because of the quite complicated nature of the physical phenomena observed in MLS, formulating exact model equations is not simple. The solution may then be intelligent control using fuzzy systems, especially Takagi-Sugeno systems^19^, artificial neural networks^20^, RBF networks^21^ or recurrent neural networks (see^22^ and the references there) to propose some data driven control approach to MLSs. Most of reported results concentrate on using artificial neural networks or fuzzy systems to modify (optimize) on-line the parameters of other controllers, such as PID and state feedback controllers in maglev levitation control.

Careful inspection of references indicates that linear controllers are mostly used, especially in practical applications. Because of this we decided to compare the controller developed in this contribution with linear controllers. All researchers stress that nonlinearities and unknown or changing parameters are the main difficulties for newly-developed controllers and nonlinear control together with intelligent control are the proper techniques to cope with this challenge. That is the state of art and the motivation to the research presented in this paper. The concept of intelligent approximation of the electromagnetic force, or rather acceleration caused by this force, is applied in the presented paper. We propose an extremely simple fuzzy model of the electromagnetic acceleration, the characteristic feature of which is a very short execution time. The proposed model ensures easy and fast inversion, i.e. calculation of the current which generates the desired acceleration. This concept is combined with the adaptive control approach, and allows continuous tunning of the fuzzy model parameters as well as other adaptive parameters substituting unknown parameters of the MLS. As MLS is not a strict-feedback system^23^ (it is in pure-feedback form) it is impossible to apply the standard adaptive backstepping approach^24^ and the novel adaptive control is tailored especially for MLS application. Using the Lyapunov approach it is proven that the errors are uniformly ultimately bounded^23^ and the guidelines for controller parameters tunning are derived.

**Figure 1:**
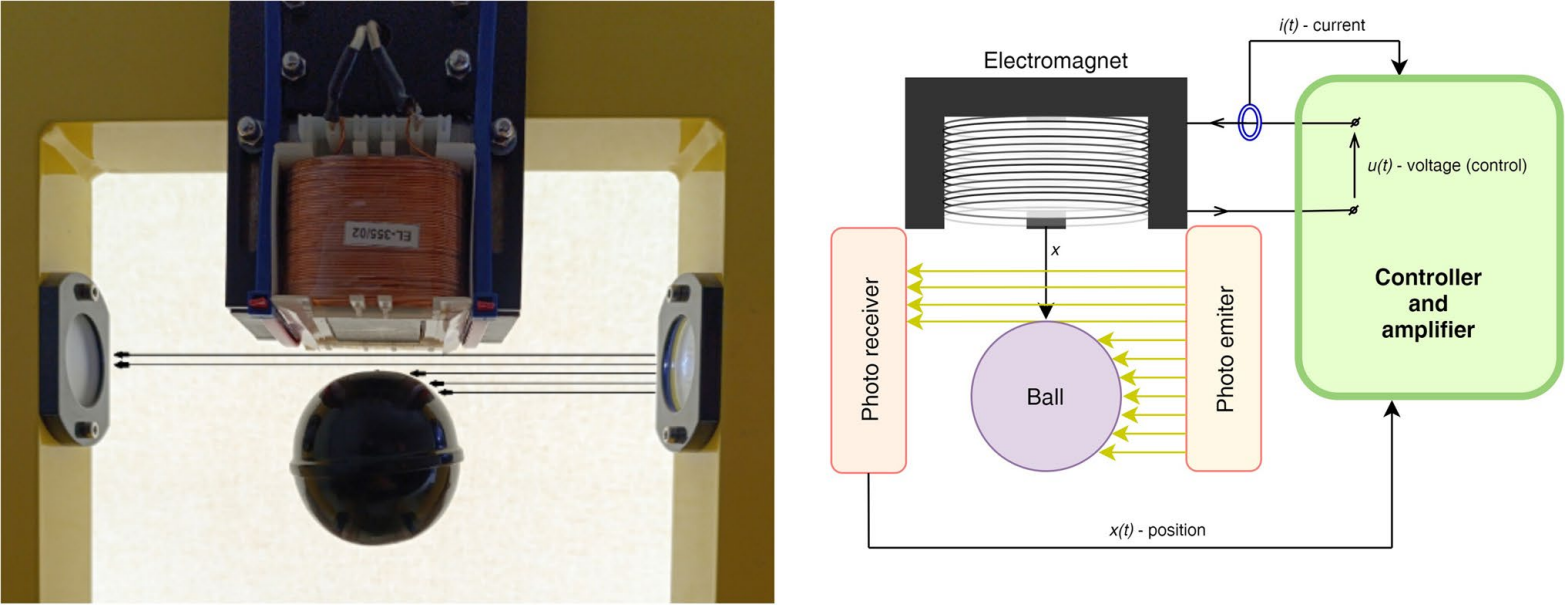
Magnetic levitation system.

The proposed controller is tested by simulations and it is implemented on a DSP board controlling a standard magnetic levitation laboratory stand. This MLs is presented in Fig. 1. The force of the electromagnet works against gravity and causes levitation of the ferromagnetic ball. The ball’s position is measured by the optical sensor (photo-emitter and photo-receiver) and is fed back to the controller which generates the coil voltage. The coil current is also measured.

The main contributions are summarized as follows.

To solve the problem of tracking a desired position trajectory by a MLS with unknown parameters:

1. A simple fuzzy model of the electromagnetic acceleration is proposed, which allows very short on-line execution time and is suitable to perform fuzzy inversion quickly and easily.
2. Novel adaptive control is derived using fuzzy inversion of the proposed electromagnetic acceleration model and on-line adaptation of fuzzy model consequents as well as other model parameters. The controller ensures sufficiently accurate tracking of any smooth desired position trajectory, which is proved using Lyapunov techniques.
3. It is proven by demonstration that the proposed solution is applicable in practice. The derived controller is investigated by numerical simulation and successful experimental verification on a typical didactic magnetic levitation laboratory stand controlled from a DSP board. Therefore it is shown that even complicated and advanced control algorithms can be demonstrated and investigated using commonly available hardware.

The paper is organized as follows. The next section is devoted to discussion and literature review of mathematical modelling of MLS. As a conclusion drawn from this discussion, the fuzzy model is proposed and the fuzzy inversion is introduced in the section ‘Fuzzy model of electromagnetic acceleration’. This section is concluded by an example demonstrating this modelling technique. Then, the tracking control problem is formulated and solved by derivation of the adaptive controller and proving its stability in the section ‘Adaptive control based on fuzzy inversion’. The main features of the derived controller are investigated in the next section by numerical experiments. Finally, as it is described in the section ‘Real plant experiments’, the derived controller is verified by implementation on a DSP board controlling a laboratory magnetic levitation stand. Section ‘Conclusions’ summarizes this study and discusses the obtained results as well as potential future research.

## Mathematical model of MLS

Each MLS, as in Fig. 1, contains an electromagnetic coil generating a magnetic field and a ferromagnetic object moving within this field. This motion changes the properties of the magnetic circuit and, together with such effects as magnetic saturation, irregular magnetic paths, non-uniform air gap, etc., is the reason that both the flux associated with the magnetic circuit and the electromagnetic force driving the moving part of the system are complex non-linear functions of the coil current and the moving body position. Modelling can be started from the equations following from the Kirchoff’s law and the second Newton’s law^25^:

1 $$\:\begin{array}{c}M\ddot{x}=Mg-{f}_{m}\left(i,x\right),\\\:\dot{\lambda\:}\left(i,x\right)=u-iR,\end{array}$$

where $\:x$ represents the position, i.e. the distance of the moving mass $\:M$ from the electromagnet (the motion down means positive velocity $\:\dot{x}$). It is assumed that $\:0<{x}_{min}<x<{x}_{max}$ in any situation, i.e. the moving body never touches the electromagnet and never exceeds minimum or maximum range of motion $\:{x}_{min}$ and $\:{x}_{max}$. The gravity affecting the body is $\:Mg$ and $\:{f}_{m}\left(i,x\right)$ is the force generated by the electromagnet. It is assumed that this force can by positive only. Although generation of electromagnetic forces in several directions is possible in some MLSs such as magnetic bearing systems^26^, in this contribution we concentrate on MLSs with just one electromagnet and ferromagnetic moving part. Such systems in which the electromagnetic force acts only in one direction are a particular challenge for the design of control algorithms and at the same time have important applications such as magnetic suspension.

The second equation describes the flux $\:\lambda\:\left(i,x\right)$ depending on the mass position and the coil current $\:i$ caused by the input voltage $\:u$. The current can be generated in the known, positive range $\:0\le\:i\le\:{i}_{max}$. The coil resistance is denoted as $\:R$. The flux can be represented as

2 $$\:\lambda\:\left(i,x\right)=L\left(x\right)i,$$

where $\:L\left(x\right)$ describes the coil inductance depending on the position of the ferromagnetic body. The function $\:L\left(x\right)$ is a decreasing function and can be selected in several ways, depending on size and geometry of the magnetic circuit and range of motion. Several exemplary models reported in references are summarized in Table 1.

**Table 1:** Models of coil inductance used in references.

Model	Reference	Parameters description
$\:L\left(x\right)={L}_{\infty\:}+\frac{{L}_{0}{x}_{0}}{x}$	^27^	$\:{L}_{\infty\:}$ - coil inductance when the ferromagnetic body is removed from the magnetic circuit $\:{L}_{\infty\:}+\:{L}_{0}$ - coil inductance when the ferromagnetic body is away by $\:{x}_{0}$ from the electromagnet $\:{L}_{\infty\:}+\:{L}_{1}$ - coil inductance when the ferromagnetic body is closest to the electromagnet $\:{a}_{n}\cdots{a}_{0},\:\alpha\:,\beta\:$ – tuned parameters
$\:L\left(x\right)={L}_{\infty\:}+{L}_{1}{e}^{-\alpha\:x}$	^28^	
$\:L\left(x\right)={L}_{\infty\:}+\frac{{L}_{1}}{1+\frac{x}{\beta\:}}$	^29^	
$\:L\left(x\right)={L}_{\infty\:}+\frac{{L}_{1}}{{\left(1+\frac{x}{\beta\:}\right)}^{2}}$	^30^	
$\:L\left(x\right)={L}_{\infty\:}+\frac{1}{{a}_{n}{x}^{n}+{a}_{n-1}{x}^{n-1}+\dots\:+{a}_{1}x+{a}_{0}}$	^31^	

The choice of the inductance model determines the relationship describing the force generated by the electromagnet^32^:

3 $$\:{f}_{m}\left(i,x\right)=-\frac{1}{2}\frac{dL}{dx}{i}^{2}$$

(positive $\:{f}_{m}\left(i,x\right)\:$ acts against the gravity, the derivative $\:\frac{dL}{dx}$ is negative as $\:L\left(x\right)$ is a decreasing function). For instance, if

4 $$\:L\left(x\right)=L_{\infty\:}+\frac{L_0x_0}x,$$

then

5 $$\:f_m\left(i,x\right)=\frac{L_0x_0}2\frac{i^2}{x^2}.$$

Formula (5) supports the assumption that the force is proportional to $\:{i}^{2}$, which is the most popular approach.

Some researchers, for instance^22^, simplify the MLS model to the extreme taking a constant value $\:L\left(x\right)=L$ in Eq. (2) and accepting

6 $$\:{f}_{m}\left(i,x\right)=K\frac{{i}^{2}}{{x}^{2}},\:\:K={\frac{1}{4}\mu\:}_{0}A{N}^{2}$$

where the parameters $\:{\mu\:}_{0},\:\:A,\:\:N$ represent the vacuum permeability, permeability area, and coil turn respectively. Such a formula for $\:{f}_{m}\left(i,x\right)$ can be linearized around a given equilibrium point $\:\left({i}_{0},\:{x}_{0}\right)$, where $\:{f}_{m}\left({i}_{0},\:{x}_{0}\right)=Mg$:

7 $$\:{f}_{m}\left(i,x\right)\approx\:{f}_{m}\left({i}_{0},\:{x}_{0}\right)+2 K\frac{{i}_{0}}{{x}_{0}^{2}}\left(i-{i}_{0}\right)-2 K\frac{{i}_{0}^{2}}{{x}_{0}^{3}}\left(x-{x}_{0}\right)$$

and this provides a linear equation of motion

8 $$\:M\ddot{x}=Mg-{f}_{m}\left(i,x\right)=-2 K\frac{{i}_{0}}{{x}_{0}^{2}}\left(i-{i}_{0}\right)+2 K\frac{{i}_{0}^{2}}{{x}_{0}^{3}}\left(x-{x}_{0}\right)=2 K\frac{{i}_{0}^{2}}{{x}_{0}^{3}}x-2 K\frac{{i}_{0}}{{x}_{0}^{2}}i.$$

On the other hand, several, far more complicated models of the electromagnetic force were also reported. Magnetic saturation and others magnetic circuit imperfections can be modelled by finite elements analysis. For instance in^33^ the model

9 $$\:{f}_{m}\left(i,x\right)=\frac{1}{2}\frac{{L}_{0}}{a{\left(1+\frac{x}{a}\right)}^{2}}{i}^{2}-\left(4{a}_{4}{x}^{3}+3{a}_{3}{x}^{2}+2{a}_{2}x+{a}_{1}\right)i$$

with experimentally selected parameters $\:{a}_{1},\:\dots\:,\:{a}_{4}$ is recommended.

Another approach^34^ allows us to model magnetic circuit saturation by making the inductance current-dependent:

10 $$\:L\left(i,x\right)=\frac{{\mu\:}_{o}A{N}^{2}}{2x+\frac{{l}_{fe}}{{\mu\:}_{r}(i,x)}}$$

where $\:{l}_{Fe}$ is the length of the flux line in the iron, while $\:{\mu\:}_{r}(i,x)$ is a factor selected to model saturation. For example

11 $$\:{\mu\:}_{r}\left(i,x\right)=\frac{{\stackrel{\sim}{\mu\:}}_{rg,0}\:}{\frac{{i}^{2}}{{i}_{sat}^{2}}+1}$$

where $\:{\stackrel{\sim}{\mu\:}}_{rg,0}$ can be interpreted as a relative magnetic permeability of iron for $\:i=0$, $\:{i}_{sat}$ as the current at which saturation has to be taken into account. Instead of formula (3), having in mind (10) we get

12 $$\:{f}_{m}\left(i,x\right)=-\frac{\partial\:}{\partial\:x}{\int\:}_{0}^{i}L\left(I,x\right)I\:dI$$

and after substituting (11):

13 $$\:{f}_{m}\left(i,x\right)=\frac{{\mu\:}_{0}A{N}^{2}{i}^{2}}{\left(2x+\frac{{l}_{Fe}}{{\stackrel{\sim}{\mu\:}}_{rg,0}\:}\right)\left(2x+\frac{{l}_{Fe}}{{\stackrel{\sim}{\mu\:}}_{rg,0}\:}+\frac{{i}^{2}}{{i}_{sat}^{2}}\frac{{l}_{Fe}}{{\stackrel{\sim}{\mu\:}}_{rg,0}\:}\right)}$$

Denoting $\:\delta\::=\frac{{l}_{Fe}}{{2\stackrel{\sim}{\mu\:}}_{rg,0}\:}$ we get

14 $$\:L\left(i,x\right)=\frac{{\mu\:}_oAN^2}{2\left(x+\delta\:+\frac{i^2}{i_{sat}^2}\delta\:\right)}=\frac{2K}{\left(x+\delta\:+\frac{i^2}{i_{sat}^2}\delta\:\right)},$$

15 $$\:{f}_{m}\left(i,x\right)=\frac{{\mu\:}_{0}A{N}^{2}}{4}\frac{{i}^{2}}{\left(x+\delta\:\right)\left(x+\delta\:+\frac{{i}^{2}}{{i}_{sat}^{2}}\delta\:\right)}\:=K\frac{{i}^{2}}{\left(x+\delta\:\right)\left(x+\delta\:+\frac{{i}^{2}}{{i}_{sat}^{2}}\delta\:\right)}.$$

If the saturation is not observed ($\:{i}_{sat}\to\:\infty\:$) the model (15) takes the form

16 $$\:{f}_{m}\left(i,x\right)=K\frac{{i}^{2}}{{\left(x+\delta\:\right)}^{2}}$$

where $\:\delta\:$ represents shift of position-force characteristics.

Summing up, selecting the right mathematical model for the electromagnetic force generated in a specific MLS is a real challenge. The model must be proposed after careful inspection of the data obtained from identification experiments performed on a specific plant. It is reasonable to assume that data-based models are more reliable, as they are able to reflect all real plant properties. Therefore, in this study, we recommend a particular fuzzy model of the electromagnetic force.

Equation (1) can be slightly modified to get

17 $$\:\dot{x}=v$$

18 $$\:\dot{v}=g-f\left(i,x\right)$$

19 $$\:L\left(x\right)\frac{di}{dt}=u-iR-\frac{\partial\:L}{\partial\:x}vi$$

where $\:f\left(i,x\right)={f}_{m}\left(i,x\right)/M$ is the acceleration due to the force $\:{f}_{m}\left(i,x\right)$ and $\:v$ is the mass velocity. We assume that the inductance corresponding to the body position $\:x$ is given by

20 $$\:L\left(x\right)={L}_{\infty\:}+{L}_{1}p\left(x\right)\:$$

where $\:p\left(x\right)$ is a known, smooth function. We assume that the constant coefficients $\:{L}_{\infty\:},\:\:{L}_{1},\:\:R,\:\:M$ are unknown, although a reasonable initial guess is possible.

Instead of modelling the force $\:{f}_{m}\left(i,x\right)$, a fuzzy model of the acceleration $\:f\left(i,x\right)$ will be used. This approach allows to exclude the unknown mass $\:M$ from the derivation.

## Fuzzy model of electromagnetic acceleration

We propose a specific fuzzy model for the acceleration $\:f\left(i,x\right)$. Two main objectives are to ensure a very short model execution time and to facilitate exact model inversion with respect to the first argument $\:i$.

**Figure 2:**
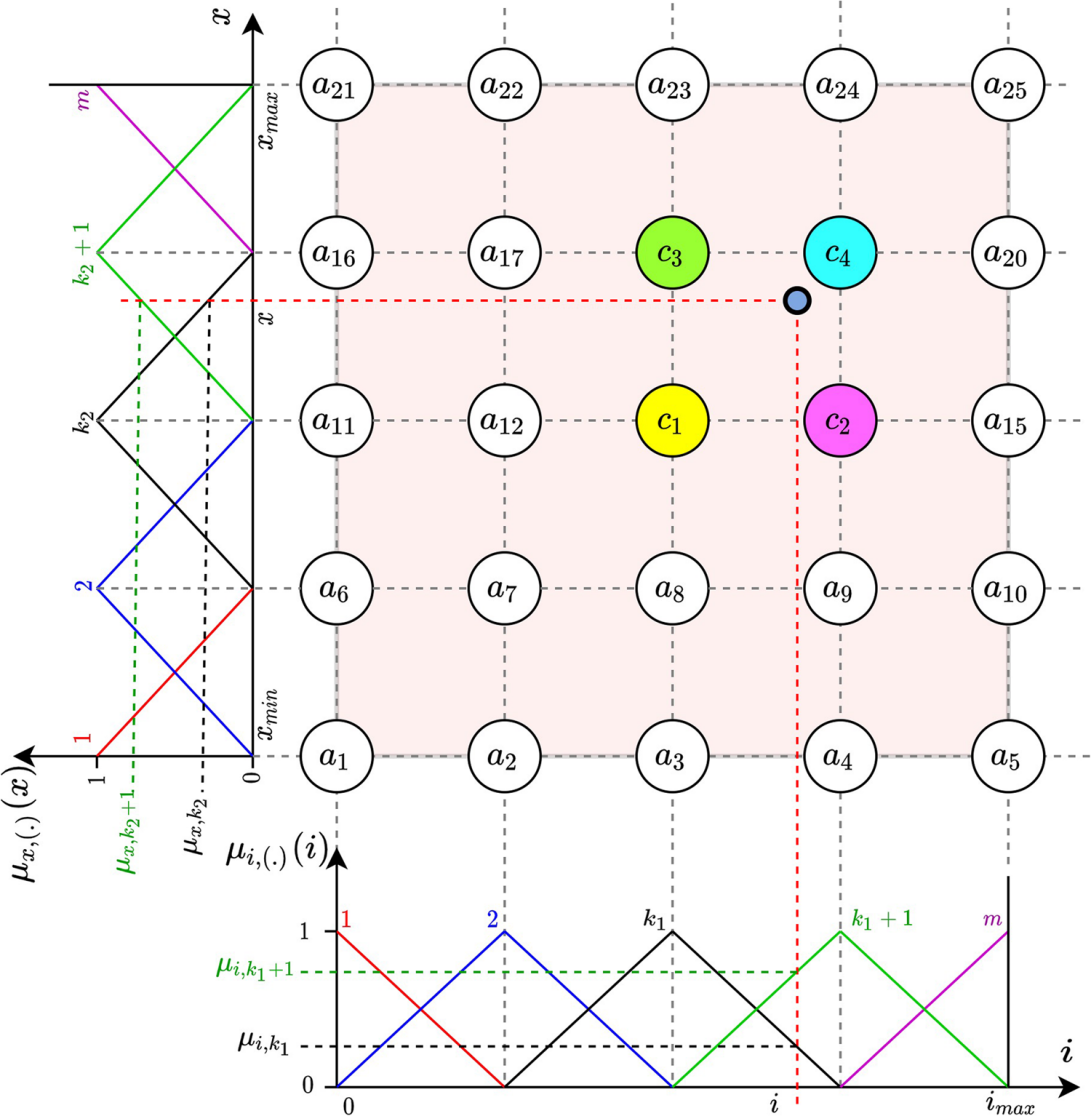
Structure of the fuzzy model of electromagnetic acceleration.

To model the acceleration $\:y=f\left(i,x\right)$ we propose a two-input fuzzy Takagi-Sugeno-Kang system with constant consequents^35^. The model structure, demonstrated in Fig. 2, is determined by the following assumptions:

1. the inputs are limited to known intervals $\:i\in\:[0,{i}_{max}]$, $\:x\in\:[{x}_{min},{x}_{max}]$,
2. for input $$\:i$$ we define $$\:m>1$$ symmetric, uniformly distributed, triangular membership functions $$\:{\mu\:}_{i,k}\left(i\right),\:\:k=1,\dots\:,m$$ such that $$\:\sum\:_{k=1}^{m}{\mu\:}_{i,k}\left(i\right)=1$$; although membership functions for input $$\:x$$ can be defined arbitrarily^36,37^, we assume the same number and type of membership functions $$\:{\mu\:}_{x,k}\left(x\right)$$$$\:\:\:k=1,\dots\:,m$$ such that $$\:\sum\:_{k=1}^{m}{\mu\:}_{x,k}\left(x\right)=1$$ for input $$\:x$$
3. the model contains $\:N={m}^{2}$ rules and the $\:j$-th rule is:

21 $$\:IF\:i\:is\:{\mu\:}_{i,{k}_{1}}\left(i\right)\:AND\:x\:is\:{\mu\:}_{x,{k}_{2}}\left(x\right)\:THEN\:y={a}_{j},\:\:{a}_{j}=const$$

where $\:A={\left[{a}_{1},\:\dots\:,\:{a}_{N}\right]}^{T}$ represents the vector of constant consequent parameters,

4. the rule firing strength for the $\:j$-th rule is calculated for any $\:i,\:x$ as

22 $$\:{\mu\:}_{j}(i,x)={\mu\:}_{i,{k}_{1}}\left(i\right)\cdot\:{\mu\:}_{x,{k}_{2}}\left(x\right)\; \;for\;\;1\le\:{k}_{1},{k}_{2}\le\:m,$$

5. the output of the model is calculated using the weighted average:

23 $$\:y(i,x)=\sum\:_{j=1}^{N}{\mu\:}_{j}(i,x){a}_{j}\:/\:\sum\:_{j=1}^{N}{\mu\:}_{j}(i,x)\:$$

This simple arrangement of membership functions causes that, for any input, at most two of them are positive at the same moment, so at most four rules generate positive rule firing strengths. Moreover, for any $\:(i,x)$

24 $$\:\sum\:_{j=1}^{N}{\mu\:}_{j}(i,x)\:=1,$$

hence,

25 $$\:y\left(i,x\right)=\sum\:_{j=1}^{N}{\mu\:}_{j}\left(i,x\right){a}_{j}\:={A}^{T}\mu\:\left(i,x\right),\:\:\:\:\:\:\:$$

where $$\:\:\mu\:\left(i,x\right):=\left[{\mu\:}_1\left(i,x\right),\:\cdots\:,\:{\mu\:}_N\left(i,x\right)\right]^T,\:\:A:=\left[a_1,\:\cdots\:,\:a_N\right]^T.\:\:$$  

The model is completely defined by the number of rules $\:{m}^{2}$, the range of inputs $\:i\in\:[0,{i}_{max}]$, $\:x\in\:[{x}_{min}\:,{x}_{max}]$, and the consequent parameters $\:A$. The distance between subsequent peak-points of membership function is

26 $$\:{{\Delta\:}}_{i}=\frac{{i}_{max}\:}{m-1},\:\:$$

27 $$\:{\:{\Delta\:}}_{x}=\frac{{x}_{max}-{x}_{min}}{m-1}$$

respectively for each input.

The output calculation from Eq. (25) can be greatly simplified and finally described by the following algorithm:

For given $$\:\left(i,x\right)$$:

1. Calculate the activated membership functions.

28 $$\:{\mu\:}_{i,k_1}\left(i\right)=1-\frac{mod\left(i-{\Delta\:}_i,{\Delta\:}_i\right)}{{\Delta\:}_i},$$

29 $$\:{\mu\:}_{i,k_1+1}\left(i\right)=1-{\mu\:}_{i,k_1}\left(i\right),$$

30 $$\:{\mu\:}_{x,k_2}\left(x\right)=1-\frac{mod\left(x-x_{min}-{\Delta\:}_x,\:\:{\Delta\:}_x\right)}{{\Delta\:}_x},$$

31 $$\:{\mu\:}_{x,{k}_{2}+1}\left(x\right)=1-{\mu\:}_{x,{k}_{2}}\left(x\right).$$

where

32 $$\:k_1=1+floor\left(\frac i{{\Delta\:}_i}\right),$$

33 $$\:k_2=1+floor\left(\frac{x-x_{min}}{{\Delta\:}_x}\:\right),$$

2. Identify four activated rules with indices $\:{j}_{1},\:\dots\:,\:{j}_{4}$ (if the rules are numbered as in Fig. 2 we have $\:{j}_{1}={k}_{1}+m\left({k}_{2}-1\right)$).
3. Calculate the output.

$$\:y=\sum\limits_{j=1}^{N}{\mu\:}_{j}{a}_{j}={\mu\:}_{i,{k}_{1}}\left(i\right)\cdot\:{\mu\:}_{x,{k}_{2}}\left(x\right)\cdot\:{a}_{{j}_{1}}+{\mu\:}_{i,{k}_{1}+1}\left(i\right)\cdot\:{\mu\:}_{x,{k}_{2}}\left(x\right)\cdot\:{a}_{{j}_{2}}+$$

34 $$\:\:\:\:\:\:\:\:\:\:\:\:\:+{\mu\:}_{i,k_1}\left(i\right)\cdot\:{\mu\:}_{x,k_2+1}\left(x\right)\cdot\:a_{j_3}+{\mu\:}_{i,k_1+1}\left(i\right)\cdot\:{\mu\:}_{x,k_2+1}\left(x\right)\cdot\:a_{j_4}.$$

The proposed fuzzy model is a perfect approximator in the sense of fuzzy systems theory^38^: for any modelling error $\:\epsilon\:$ there exists a number of rules $\:N={m}^{2}$ such that for any $\:i\in\:[0,{i}_{max}]$, $\:x\in\:[{x}_{min},{x}_{max}]$ the electromagnetic acceleration is modeled with the prescribed accuracy $\:\left|f\left(i,x\right)-{A}^{T}\mu\:\left(i,x\right)\right|\le\:\epsilon\:$. For our purposes a much weaker property of the proposed fuzzy model is used: for a given number of membership functions (i.e. for a given number of rules $\:N={m}^{2}$) there exists a vector of parameters $\:A$, such that the modelling error is bounded: for any $\:i\in\:[0,{i}_{max}]$, $\:x\in\:[{x}_{min},{x}_{max}]$

35 $$\:f\left(i,x\right)=A^T\mu\:\left(i,x\right)+\epsilon\:\left(i,x\right),\:\:\:\left|\epsilon\:\left(i,x\right)\right|\leq\:{\epsilon\:}_o<\infty\:.$$

Moreover, in the adaptive control algorithm which will be developed in the next section the parameters $\:A$ will be substituted by adaptive parameters, therefore, it is not necessary to know either $\:A$ or $\:{\epsilon\:}_{o}\:$in advance. All we need is to know that such $\:A$ and $\:{\epsilon\:}_{o}$ exist and to be able to propose an initial guess $\:\stackrel{-}{A}$ for $\:A$, which can be easily achieved by initial tunning of the fuzzy model. Widely available software offers ready procedures for initialization and tunning of the proposed model. For instance in Matlab the model can be initialized by procedure *genfis* with option *GridPartition* and trained by *lsqlin.*

Generally, exact inversion of a fuzzy model $\:F(u,x)$ is understood as a numerical procedure which provides, for any $\:x$ in the domain and any $\:y$ in the range of $\:F(u,x)$, one (maybe selected from many possible) value $\:u={u}_{y}$ such that $\:F\left({u}_{y},x\right)=y$.

Several approaches to the fuzzy system inversion problem were reported, which can be divided into two groups – approximate and exact methods. Approximate techniques search for the solution iteratively, using the Newtons method^39^ or more complex global search tools such as genetic algorithms^40^. The advantage of the approximate methods is the lack of limitation on the structure of the inverted fuzzy model. A disadvantage, however, that may eliminate the possibility of using these techniques in real time, is the extended time of searching for a solution, especially in the case of hybrid methods combining global search with fine-tuning. Exact inversion methods can be applied to linguistic fuzzy models^41^ as well as to Takagi-Sugeno-Kang fuzzy systems with constant consequents^42^ or linear consequents^43^. Some of them are also limited by high computational complexity.

In this contribution the concept of fuzzy inversion proposed in^36,37^ is used. This approach offers some advantages, such as: the ability to deal with non-one-to-one mappings, very short execution time when executed by a digital controller, and implementation as a sequence of typical signal processing operations. Although the results concerning the fuzzy inversion algorithm were presented in^36,37^, we recollect the main points to make it easier to understand and to preserve consistency of notation.

For the presented fuzzy model of electromagnetic acceleration, fuzzy system inversion calculates, for given $\:x$ and $\:y\:$from the range of $\:{A}^{T}\mu\:\left({i}_{y},x\right)$ over $\:[0,{i}_{max}]$, the current $\:i={i}_{y}$ which generates prescribed model output $\:y={A}^{T}\mu\:\left({i}_{y},x\right)$. Assume that $\:x$ activates membership functions $\:{\mu\:}_{x,{k}_{2}},\:\:{\mu\:}_{x,{k}_{2}+1}$. As described in^36,37^, the method of calculation is based on a simple observation that, assuming that the current value $\:{i}_{y}$ activates the membership functions $\:{\mu\:}_{i,{k}_{1}},\:\:{\mu\:}_{i,{k}_{1}+1}$, we know that four rules $\:{j}_{1},\:\dots\:,{j}_{4}$ are activated and we get from (29,31,34):

$$\:y={\mu\:}_{i,{k}_{1}}\left({i}_{y}\right)\left[{\mu\:}_{x,{k}_{2}}\left(x\right)\cdot\:{a}_{{j}_{1}}+{\mu\:}_{x,{k}_{2}+1}\left(x\right)\cdot\:{a}_{{j}_{3}}\right]+{\mu\:}_{i,{k}_{1}+1}\left({i}_{y}\right)\left[{\mu\:}_{x,{k}_{2}}\left(x\right)\cdot\:{a}_{{j}_{2}}+{\mu\:}_{x,{k}_{2}+1}\left(x\right)\cdot\:{a}_{{j}_{4}}\right]=$$

$$\:={\mu\:}_{i,{k}_{1}}\left({i}_{y}\right)\left[{\mu\:}_{x,{k}_{2}}\left(x\right)\cdot\:{a}_{{j}_{1}}-{\mu\:}_{x,{k}_{2}}\left(x\right)\cdot\:{a}_{{j}_{2}}+{\mu\:}_{x,{k}_{2}+1}\left(x\right)\cdot\:{a}_{{j}_{3}}-{\mu\:}_{x,{k}_{2}+1}\left(x\right)\cdot\:{a}_{{j}_{4}}\right]+$$

36 $$\:+{\mu\:}_{x,{k}_{2}}\left(x\right)\cdot\:{a}_{{j}_{2}}+{\mu\:}_{x,{k}_{2}+1}\left(x\right)\cdot\:{a}_{{j}_{4}}$$

and hence, the value of $\:{\mu\:}_{i,{k}_{1}}\left({i}_{y}\right)$ should be

37 $$\:{\mu\:}_{i,{k}_{1}}^{*}={\mu\:}_{i,{k}_{1}}\left({i}_{y}\right)=\frac{y-{\mu\:}_{x,{k}_{2}}\left(x\right)\cdot\:{a}_{{j}_{2}}-{\mu\:}_{x,k+1\:}\left(x\right)\cdot\:{a}_{{j}_{4}}}{{\mu\:}_{x,{k}_{2}}\left(x\right)\cdot\:{a}_{{j}_{1}}-{\mu\:}_{x,{k}_{2}}\left(x\right)\cdot\:{a}_{{j}_{2}}+{\mu\:}_{x,{k}_{2}+1}\left(x\right)\cdot\:{a}_{{j}_{3}}-{\mu\:}_{x,{k}_{2}+1}\left(x\right)\cdot\:{a}_{{j}_{4}}}$$

If the calculated value $\:{\mu\:}_{i,{k}_{1}}^{*}$ is admissible ($\:0<{\mu\:}_{i,{k}_{1}}^{*}\le\:1)$, we get that the current:

38 $$\:{i}_{y,{k}_{1}}={{\Delta\:}}_{i}\left({k}_{1}-{\mu\:}_{i,{k}_{1}}^{*}\right)$$

fulfils the required condition $\:{A}^{T}\mu\:\left({i}_{y,{k}_{1}},x\right)=y.\:$If not, our assumption that $\:{i}_{y}$ activates membership functions $\:{\mu\:}_{i,{k}_{1}},\:\:{\mu\:}_{i,{k}_{1}+1}$ was not correct. In any case, we have to test all other membership functions instead of $\:{\mu\:}_{i,{k}_{1}}$ as the equation $\:y={A}^{T}\mu\:\left({i}_{y},x\right)$ may possess multiple solutions. After repeating the presented procedure for $\:{k}_{1}=1,\:\dots\:,\:m-1$ we get a set of $\:q+1$ current values $\:{I}_{y}=\{{i}_{y,{k}_{j}},\:{i}_{y,{k}_{j+1}},\:\dots\:{i}_{y,{k}_{j+q}}\}$, each of them generating the desired model output $\:y={A}^{T}\mu\:\left({i}_{y,{k}_{j+l}},x\right)=y,\:\:l=0,\:\dots\:,q$. If $\:q>0$ we select the smallest value from the set $\:{I}_{y}$. If $\:q=0$ we get the unique solution.

Both procedures – creating the fuzzy model and the fuzzy inversion are illustrated by the following example.

**Figure 3:**
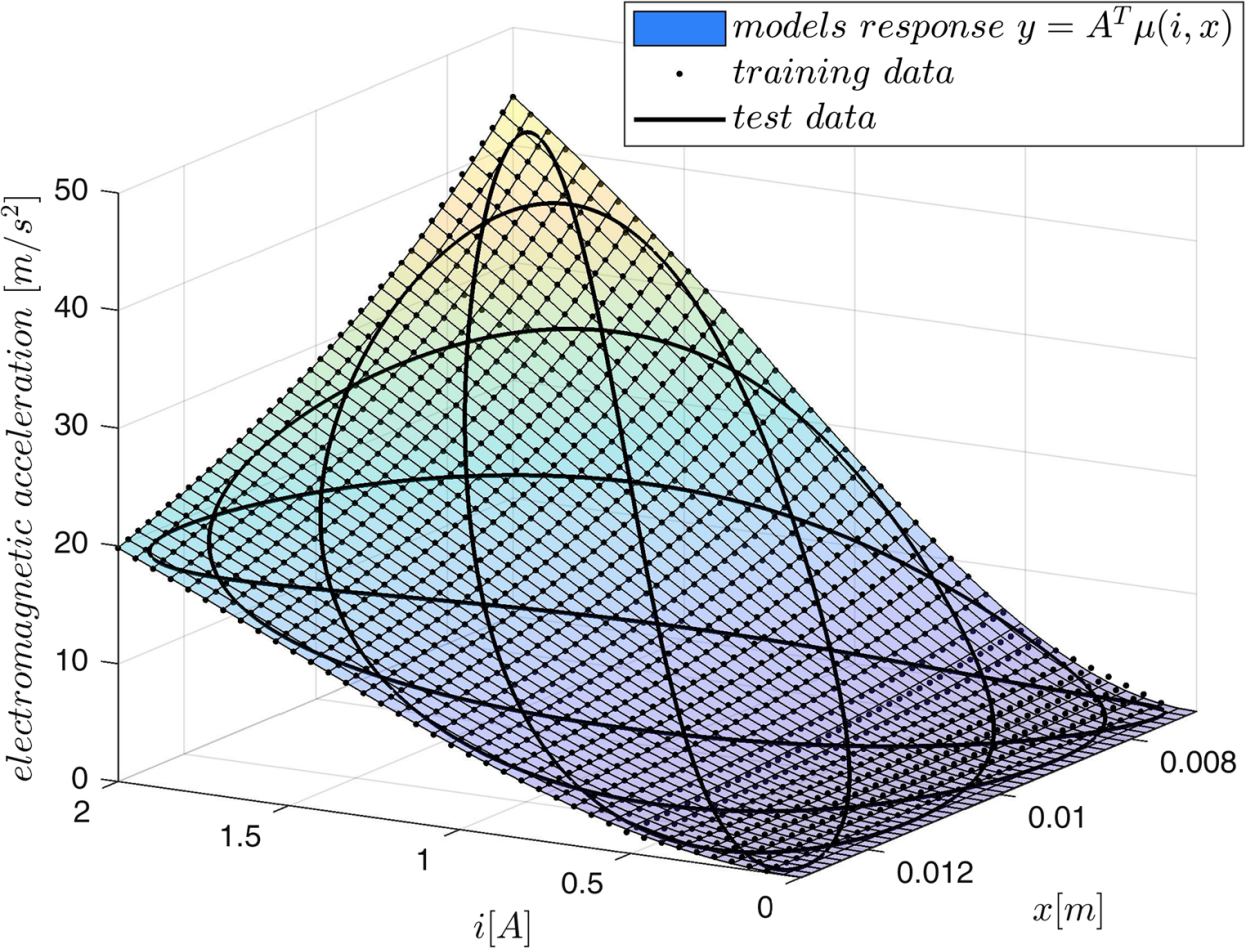
Acceleration surface obtained from the model, training data (dots) and the testing trajectory.

**Figure 4:**
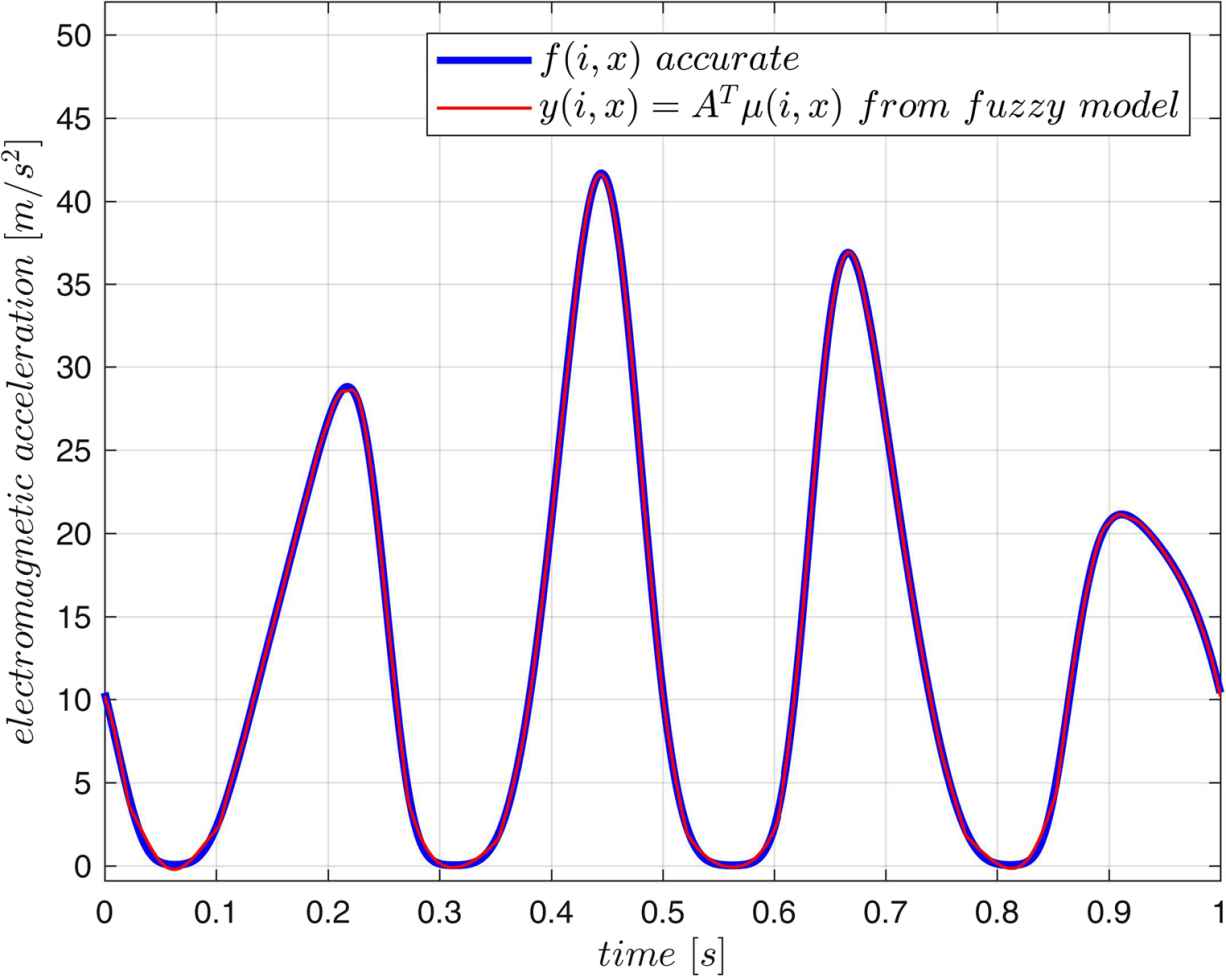
Time history corresponding to the testing trajectory obtained from the fuzzy model and from the exact formula – both curves almost overlap.

Example 1: We assume that the accurate electromagnetic acceleration surface is given by the force model (15) assuming :

39 $$\:f\left(i,x\right)=1.8\cdot\:{10}^{-3}\frac{{i}^{2}}{\left(x+0.002\right)\left(x+0.002+0.002\frac{{i}^{2}}{{0.95}^{2}}\right)}$$

over $\:0\le\:i\le\:2\:\:\left[A\right]$ and $\:0.007\le\:x\le\:0.013\:\:\left[m\right]$. For $\:40\times\:40$ uniformly distributed values of current and position the exact outputs from (39) were collected to form 1600 training triples $\:\left(i,x,\:f\left(i,x\right)\right)$. For $\:m=5$ membership functions the 25-rule fuzzy model was created and trained by the procedure *lsqlin* starting from zeros as initial values of 25 parameters $\:A$. The surface obtained from the model together with the training data and the testing trajectory $\:R$ given by:

40 $$\:\begin{array}{c}x\left(t\right)=2.9\cdot\:{10}^{-3}\text{sin}\left(10\pi\:t-\pi\:\right)\left[m\right]\\\:i\left(t\right)=0.95\text{sin}\left(8\pi\:t-pi\right)+1\:\:\:\:\:\:\left[A\right]\:\end{array}\:0\le\:t<1\left[s\right]$$

are presented in Fig. 3. The output of the model for the testing trajectory is compared with exact values obtained from (39) in Fig. 4. The maximum modelling error over the testing data $\:R$ for the 25-rule fuzzy model is not bigger then $\:0.7\:[m/{s}^{2}]$. This error depends on the number of rules as it is demonstrated in Table 2.

**Table 2:** Maximum errors of fuzzy models and fuzzy inversion.

$N={m}^{2}$	$\:9$	$\:16$	$\:25$	$\:49$	$\:100$	$\:225$
maximum absolute error of fuzzy model$\::$						
$\max\limits_{\mathit(i\mathit(t\mathit)\mathit,x\mathit(t\mathit)\mathit)\mathit\in R}\vert f(i(t),x(t))-y(i(t),x(t))\vert\lbrack m/s^2\rbrack$	$\:2.02$	$\:0.87$	$\:0.68$	$\:0.49$	$\:0.18$	0.08
$\frac{{\displaystyle\max\limits_{\mathit(i\mathit(t\mathit)\mathit,x\mathit(t\mathit)\mathit)\mathit\in R}}\vert\mathrm f(\mathrm i(\mathrm t),\mathrm x(\mathrm t))-\mathrm y(\mathrm i(\mathrm t),\mathrm x(\mathrm t))\vert}{{\displaystyle\max\limits_{\mathit(i\mathit(t\mathit)\mathit,x\mathit(t\mathit)\mathit)\mathit\in R}}\vert\mathrm f(\mathrm i(\mathrm t),\mathrm x(\mathrm t))\vert}\cdot100\;\lbrack\%\rbrack$	$\:4.75$	$\:2.05$	$\:1.60$	$\:1.15$	$\:0.42$	0.19
maximum absolute error of fuzzy inversion:						
$\max\limits_{(\mathrm f(\mathrm t),\mathrm x(\mathrm t))\in\overset={\mathrm R}\;}\vert i(t)-i_f(f(t),x(t))\vert\lbrack A\rbrack$	$\:0.11$	$\:0.063$	$\:0.049$	$\:0.041$	$\:0.026$	$\:0.019$
$\frac{\displaystyle\max\limits_{\mathit(f\mathit(t\mathit)\mathit,x\mathit(t\mathit)\mathit)\mathit\in\overset{\mathit=}R\;}\vert i(t)-i_f(f(t),x(t))\vert}{\displaystyle\max\limits_{0\leq\mathrm t\leq1}\vert i(t)\vert}\cdot100\;\lbrack\%\rbrack$	$\:5.64$	$\:3.23$	$\:2.51$	$\:2.10$	$\:1.33$	$\:0.97$

**Figure 5:**
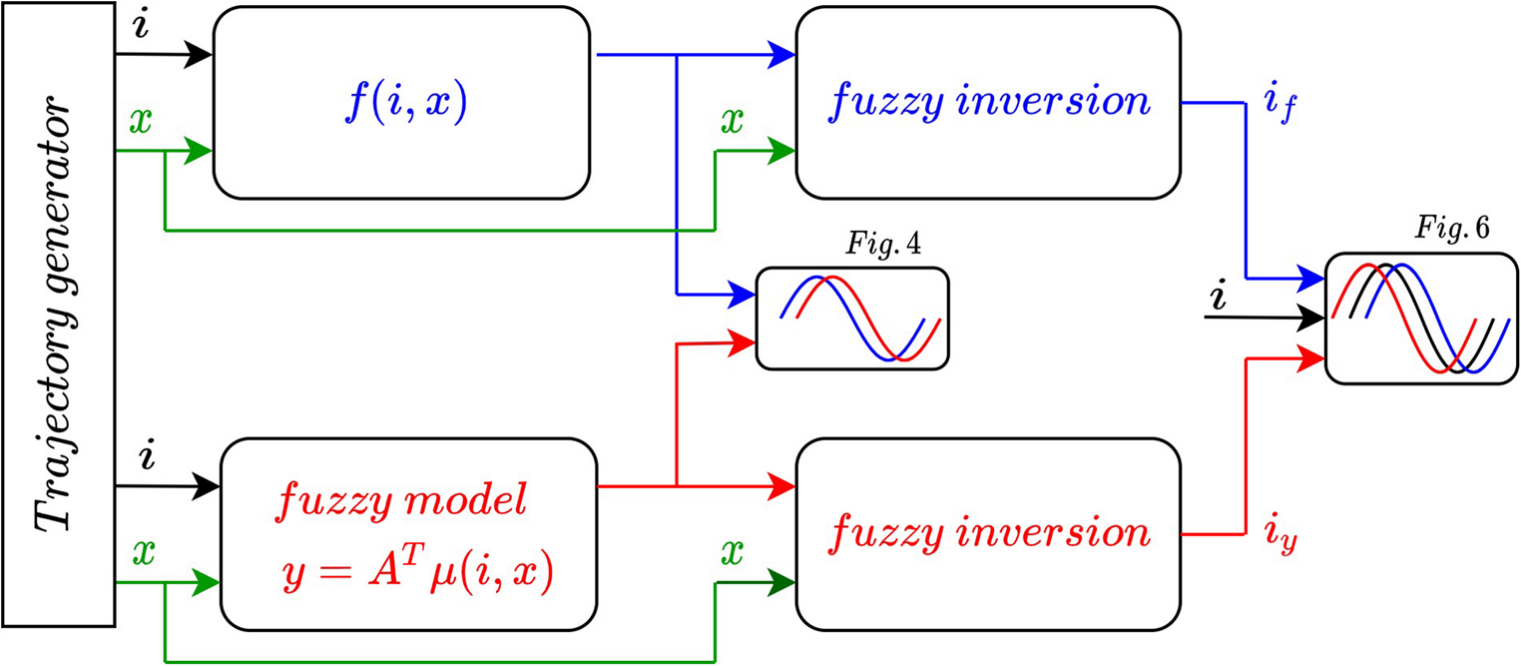
Methodology of testing fuzzy inversion accuracy.

**Figure 6:**
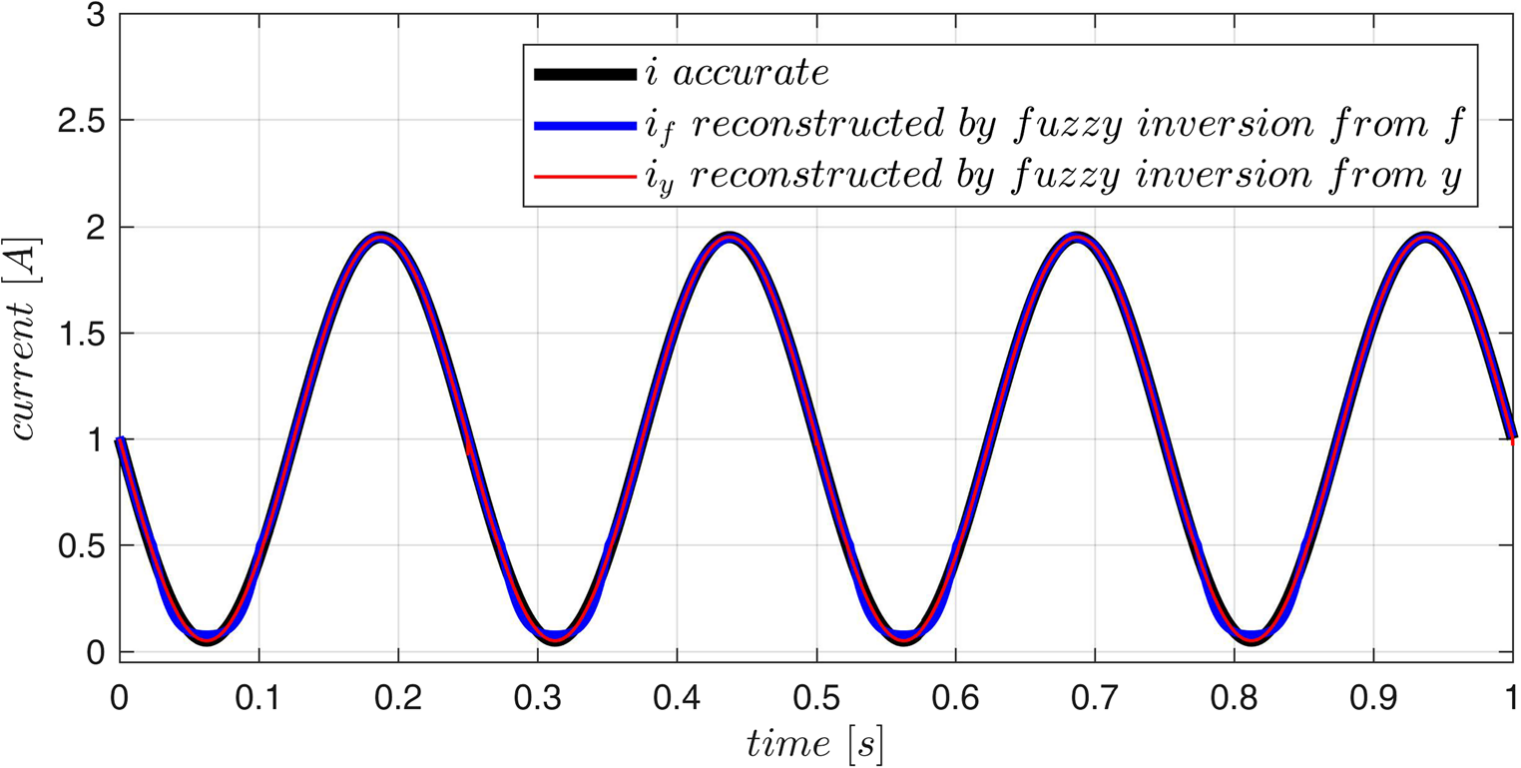
Accurate current (black) and results of fuzzy inversion.

Next, the described fuzzy inversion procedure was tested. Methodology of the test is illustrated in Fig. 5 and the results are presented in Fig. 6. For two independent time-trajectories (40) (the testing data $\:R$) the accurate acceleration $\:f(i,x)$ was calculated from (39). The set $\:\stackrel{-}{R}=\{\left(f,x\right):f=f(i\left(t\right),x\left(t\right),\:x=x\left(t\right),\:\:(i\left(t\right),x\left(t\right))\in\:R\}$ forms the test data-set for the fuzzy inversion. The exact acceleration $\:f(i,x)$ was compared with the approximated one $\:y(i,x)$ delivered by the tuned fuzzy model (Fig. 4). If the fuzzy inversion algorithm operates on the acceleration data $\:y,$ which approximates the exact acceleration $\:f$ and is provided by the fuzzy model, it calculates the current $\:i$ at the model input accurately: $\:{i}_{y}=i$ (Fig. 6). This is a characteristic feature of exact fuzzy inversion. If the same algorithm operates on the exact acceleration $\:f$, it approximates the actual current $\:i$ by the calculated value $\:{i}_{f}$. The error of this approximation depends on the accuracy (number of rules) of the fuzzy model as it is presented in Table 2.

## Adaptive control based on fuzzy inversion

Let us consider the MLS described by Eqs. (17–20). There exists a fuzzy model of the electromagnetic acceleration surface $\:y={A}^{T}\mu\:\left(i,x\right)$ described in the previous section and fulfilling condition (35). The initial guess for the unknown, constant parameters $\:A$ is denoted by $\:\stackrel{-}{A}$. We also assume that constant parameters $\:{B}^{T}=[{L}_{\infty\:},R,{L}_{1}]$ are unknown and $\:\stackrel{-}{B}$ denotes the initial estimation of $\:B$. Unknown parameters $\:A$ and $\:B$ will be substituted by adaptive parameters $\:\widehat{A}$ and $\:\widehat{B}$, and the errors of adaptation will be denoted by:

41 $$\:\stackrel{\sim}{A}=A-\widehat{A},\:\:\:\:\stackrel{\sim}{B}=B-\widehat{B}.$$

The control aim is to follow a smooth desired position trajectory $\:{x}_{d}\left(t\right)$ sufficiently close. During the derivation of the controller we will define desired trajectories for velocity and current: $\:{v}_{d}$ and $\:{i}_{d}$ respectively. Let us denote corresponding tracking errors by

42 $$\:{e}_{x}={x}_{d}-x, \;\;{e}_{v}={v}_{d}-v ,\;\;{e}_{i}={i}_{d}-i$$

The plan of the derivation is to design a control law for the control input $\:u$ and adaptive laws for the adaptive parameters $\:\widehat{A}$ and $\:\widehat{B}$, such that all errors $\:{e}_{x},\:{e}_{v},\:{e}_{i},\:\stackrel{\sim}{A},\:\stackrel{\sim}{B}\:$ are uniformly ultimately bounded (UUB)^23^ to an attraction set containing 0, and this set can be narrowed by a proper choice of design parameters. Therefore we consider a Lyapunov function

43 $$\:V=\frac{1}{2}{e}_{x}^{2}+\frac{1}{2}{e}_{v}^{2}+\frac{1}{2}L\left(x\right){e}_{i}^{2}+\frac{1}{2}{\stackrel{\sim}{A}}^{T}{{\Gamma\:}}_{\text{A}}^{-1}\stackrel{\sim}{A}+\frac{1}{2}{\stackrel{\sim}{B}}^{T}{{\Gamma\:}}_{\text{B}}^{-1}\stackrel{\sim}{B}$$

with positive definite matrices $\:{{\Gamma\:}}_{A}$ and $\:{{\Gamma\:}}_{B}$ of proper dimensions. Higher eigenvalues of the matrices $\:{{\Gamma\:}}_{A}$ and $\:{{\Gamma\:}}_{B}$ will result in faster adaptation of parameters $\:\widehat{A}$ and $\:\widehat{B}$. We introduce the derivative of the Lyapunov function along the system trajectories

44 $$\:\dot{V}={e}_{x}{\dot{e}}_{x}+{e}_{v}{\dot{e}}_{v}+{e}_{i}L\left(x\right){\dot{e}}_{i}+\frac{1}{2}\frac{dL}{dx}v{e}_{i}^{2}+{\stackrel{\sim}{A}}^{T}{{\Gamma\:}}_{\text{A}}^{-1}\dot{\stackrel{\sim}{A}}+{\stackrel{\sim}{B}}^{T}{{\Gamma\:}}_{\text{B}}^{-1}\dot{\stackrel{\sim}{B}}$$

and the plan is to demonstrate that this derivative is negative outside of a certain compact set around 0. The aim is to represent $\:\dot{V}$ as a negative definite function of state variables $\:{e}_{x},\:{e}_{v},\:{e}_{i},\:\stackrel{\sim}{A},\:\stackrel{\sim}{B}\:$ plus, eventually, a positive bounded component as small as possible. There are various technical tricks to achieve this aim and the presented derivation is one of several possible.

After substitution of error derivatives expressed with the use of (17–19) as

45 $$\:{\dot e}_x={\dot x}_d-\dot x={\dot x}_d-v,$$

46 $$\:{\dot e}_v={\dot v}_d-\dot v={\dot v}_d-g+f\left(i,x\right),$$

47 $$\:L\left(x\right){\dot{e}}_{i}=L\left(x\right)\frac{{di}_{d}}{dt}-L\left(x\right)\frac{di}{dt}=L\left(x\right)\frac{{di}_{d}}{dt}-u+iR+\frac{dL}{dx}vi$$

into (44) we get

48 $$\:\dot{V}={e}_{x}({\dot{x}}_{d}-v)+{e}_{v}\left[{\dot{v}}_{d}-g+f\left(i,x\right)\right]+{e}_{i}\left(L\left(x\right)\frac{{di}_{d}}{dt}-L\left(x\right)\frac{di}{dt}\:\:+\frac{1}{2}\frac{dL}{dx}v{e}_{i}\right)+{\stackrel{\sim}{A}}^{T}{{\Gamma\:}}^{-1}\dot{\stackrel{\sim}{A}}+{\stackrel{\sim}{B}}^{T}{{\Gamma\:}}_{\text{B}}^{-1}\dot{\stackrel{\sim}{B}}$$

Using parameterization of the inductance described in Eq. (20) provides

49 $$\:\dot{V}={e}_{x}({\dot{x}}_{d}-v)+{e}_{v}\left[{\dot{v}}_{d}-g+f\left(i,x\right)\right]+{e}_{i}\left[{L}_{\infty\:}\frac{{di}_{d}}{dt}-u+iR+{L}_{1}\left[p\left(x\right)\frac{d{i}_{d}}{dt}+\frac{dp}{dx}v\left(i+\frac{1}{2}{e}_{i}\right)\:\right]\right]+{\stackrel{\sim}{+A}}^{T}{{\Gamma\:}}^{-1}\dot{\stackrel{\sim}{A}}+{\stackrel{\sim}{B}}^{T}{{\Gamma\:}}_{\text{B}}^{-1}\dot{\stackrel{\sim}{B}}$$

Expression (49) can be shortened to

50 $$\:\dot{V}={e}_{x}({\dot{x}}_{d}-v)+{e}_{v}\left[{\dot{v}}_{d}-g+f\left(i,x\right)\right]+{e}_{i}\left[{B}^{T}\xi\:-u\right]+{\stackrel{\sim}{A}}^{T}{{\Gamma\:}}_{\text{A}}^{-1}\dot{\stackrel{\sim}{A}}+{\stackrel{\sim}{B}}^{T}{{\Gamma\:}}_{\text{B}}^{-1}\dot{\stackrel{\sim}{B}}$$

where

51 $$\:\xi\:=\left[\frac{di_d}{dt},\:\:i,\:\:p\left(x\right)\frac{di_d}{dt}+\frac{dp}{dx}v\left(i+\frac12e_i\right)\:\right]^T.$$

After plugging in $\:v={v}_{d}-{e}_{v}$ we get

52 $$\:\dot V=e_x\left({\dot x}_d-v_d+e_v\right)+e_v\left[{\dot v}_d-g+f\left(i,x\right)\right]+e_i\left[B^T\xi\:-u\right]+\widetilde A^T{\Gamma\:}_\text{A}^{-1}\dot{\widetilde A}+\widetilde B^T{\Gamma\:}_\text{B}^{-1}\dot{\widetilde B}.$$

Virtual controls $\:{v}_{d}$, $\:{i}_{d}$and the physical control $\:u$ will be used to cancel unnecessary nonlinearities and to introduce negative definite components, while adaptive laws will eliminate components containing unknown parameters. Let us start with the first component in (52). Selection

53 $$\:v_d={\dot x}_d+k_xe_x,$$

where $\:{k}_{x}>0$ is a design parameter (higher values of $\:{k}_{x}$ will cause faster convergence of $\:{e}_{x}$), results in

54 $$\:\dot{V}=-{k}_{x}{e}_{x}^{2}+{e}_{v}\left[{e}_{x}+{\dot{v}}_{d}-g+f\left(i,x\right)\right]+{e}_{i}\left[{B}^{T}\xi\:-u\right]+{\stackrel{\sim}{A}}^{T}{{\Gamma\:}}_{\text{A}}^{-1}\dot{\stackrel{\sim}{A}}+{\stackrel{\sim}{B}}^{T}{{\Gamma\:}}_{\text{B}}^{-1}\dot{\stackrel{\sim}{B}}$$

so the negative component $\:-{k}_{x}{e}_{x}^{2}$ appears.

Let us analyse the second component in (54). Let us observe that the electromagnetic acceleration can be represented as

$$\:f\left(i,x\right)={A}^{T}\mu\:\left(i,x\right)+\epsilon\:={A}^{T}\mu\:\left(i,x\right)+\epsilon\:+{A}^{T}\mu\:\left({i}_{d},x\right)-{A}^{T}\mu\:\left({i}_{d},x\right)=$$

$$\:={\widehat{A}}^{T}\mu\:\left({i}_{d},x\right)+{\stackrel{\sim}{A}}^{T}\mu\:\left({i}_{d},x\right)+{A}^{T}\left[\mu\:\left(i,x\right)-\mu\:\left(i+{e}_{i},x\right)\right]+\epsilon\:=$$

$$\:={\widehat{A}}^{T}\mu\:\left({i}_{d},x\right)+{\stackrel{\sim}{A}}^{T}\mu\:\left({i}_{d},x\right)+{\widehat{A}}^{T}\left[\mu\:\left(i,x\right)-\mu\:\left(i+{e}_{i},x\right)\right]+{\stackrel{\sim}{A}}^{T}\left[\mu\:\left(i,x\right)-\mu\:\left(i+{e}_{i},x\right)\right]+\epsilon\:=$$

55 $$\:={\widehat{A}}^{T}\mu\:\left({i}_{d},x\right)+{\widehat{A}}^{T}\left[\mu\:\left(i,x\right)-\mu\:\left(i+{e}_{i},x\right)\right]+{\stackrel{\sim}{A}}^{T}\mu\:\left(i,x\right)+\epsilon\:.$$

Hence, after substitution of (55) into (54) we have

56 $$\:\dot{V}=-{k}_{x}{e}_{x}^{2}+{e}_{v}\left[{e}_{x}+{\dot{v}}_{d}-g+{\widehat{A}}^{T}\mu\:\left({i}_{d},x\right)\right]+{e}_{v}{\widehat{A}}^{T}\left[\mu\:\left(i,x\right)-\mu\:\left(i+{e}_{i},x\right)\right]+{{e}_{v}\stackrel{\sim}{A}}^{T}\mu\:\left(i,x\right)+{+e}_{i}\left[{B}^{T}\xi\:-u\right]+{\stackrel{\sim}{A}}^{T}{{\Gamma\:}}_{\text{A}}^{-1}\dot{\stackrel{\sim}{A}}+{\stackrel{\sim}{B}}^{T}{{\Gamma\:}}_{\text{B}}^{-1}\dot{\stackrel{\sim}{B}}+{e}_{v}\epsilon\:.$$

To create the possibility of shortening component $\:{e}_{v}{\widehat{A}}^{T}\left[\mu\:\left(i,x\right)-\mu\:\left(i+{e}_{i},x\right)\right]$ by the control $\:u$ we can write down (56) as

57 $$\:\dot{V}=-{k}_{x}{e}_{x}^{2}+{e}_{v}\left[{e}_{x}+{\dot{v}}_{d}-g+{\widehat{A}}^{T}\mu\:\left({i}_{d},x\right)\right]+{e}_{i}\left[{B}^{T}\xi\:-u+\frac{{e}_{v}{\widehat{A}}^{T}\left[\mu\:\left(i,x\right)-\mu\:\left(i+{e}_{i},x\right)\right]}{{e}_{i}}\right]+{{e}_{v}\stackrel{\sim}{A}}^{T}\mu\:\left(i,x\right)++{\stackrel{\sim}{A}}^{T}{{\Gamma\:}}_{\text{A}}^{-1}\dot{\stackrel{\sim}{A}}+{\stackrel{\sim}{B}}^{T}{{\Gamma\:}}_{\text{B}}^{-1}\dot{\stackrel{\sim}{B}}+{e}_{v}\epsilon\:.$$

The value $\:{i}_{d}$ will be calculated by inversion of the fuzzy model $\:{\widehat{A}}^{T}\mu\:\left(i,x\right)$, according to the Eq. 

58 $$\:{\widehat{A}}^{T}\mu\:\left({i}_{d},x\right)=-{e}_{x}-{\dot{v}}_{d}+g-{k}_{v}{e}_{v},$$

where $\:{k}_{v}>0$ is a design parameter - higher values of $\:{k}_{v}$ will cause faster convergence of $\:{e}_{v}$. The control $\:u$ is selected to satisfy

59 $$\:u={k}_{i}{e}_{i}+{\widehat{B}}^{T}\xi\:+\frac{{e}_{v}{\widehat{A}}^{T}\left[\mu\:\left(i,x\right)-\mu\:\left(i+{e}_{i},x\right)\right]}{{e}_{i}},$$

where $\:{k}_{i}>0$ is a design parameter - higher values of $\:{k}_{i}$ will cause faster convergence of $\:{e}_{i}$. Finally, having in mind that for constant parameters $\:A,B$

60 $$\:\dot{\widetilde A}=-\dot{\widehat A},\;\;\;\:\dot{\widetilde B}=-\dot{\widehat{B,}}$$

after plugging in (58–60) into (57) we get

61 $$\:\dot{V}=-{k}_{x}{e}_{x}^{2}-{k}_{v}{e}_{v}^{2}-{k}_{i}{e}_{i}^{2}+{\stackrel{\sim}{A}}^{T}\left({e}_{v}\mu\:\left(i,x\right)-{{\Gamma\:}}_{\text{A}}^{-1}\dot{\widehat{A}}\right)+{\stackrel{\sim}{B}}^{T}\left({e}_{i}\xi\:-{{\Gamma\:}}_{\text{B}}^{-1}\dot{\widehat{B}}\right)+{e}_{v}\epsilon\:.$$

We use robust adaptive control laws for $\:\widehat{A}$ and $\:\widehat{B}$

62 $$\:\dot{\widehat{A}}={{\Gamma\:}}_{A}\left({e}_{v}\mu\:\left(i,x\right)-{\sigma\:}_{A}\left(\widehat{A}-\stackrel{-}{A}\right)\right)$$

63 $$\:\dot{\widehat{B}}={{\Gamma\:}}_{B}\left({e}_{i}\xi\:\left(i,x\right)-{\sigma\:}_{B}\left(\widehat{B}-\stackrel{-}{B}\right)\right)\:\:\:$$

with positive definite matrices $\:{{\Gamma\:}}_{A}$, $\:{{\Gamma\:}}_{B}$ and small positive “leakage parameters” $\:{\sigma\:}_{A}$, $\:{\sigma\:}_{B}$. After substituting the adaptive laws (62) and (63) into (61) we get

64 $$\:\dot{V}=-{k}_{x}{e}_{x}^{2}-{k}_{v}{e}_{v}^{2}-{k}_{i}{e}_{i}^{2}+{\stackrel{\sim}{A}}^{T}{\sigma\:}_{A}\left(\widehat{A}-\stackrel{-}{A}\right)+{\stackrel{\sim}{B}}^{T}{\sigma\:}_{B}\left(\widehat{B}-\stackrel{-}{B}\right)+{e}_{v}\epsilon\:.$$

Let us denote

65 $$\:{\widehat{A}}_{o}:=\widehat{A}-\stackrel{-}{A},\:\:{A}_{o}:=A-\stackrel{-}{A},\:\:{\stackrel{\sim}{A}}_{o}={A}_{o}-{\widehat{A}}_{o}$$

and notice that

66 $$\:{\stackrel{\sim}{A}}_{o}={A}_{o}-{\widehat{A}}_{o}=A-\stackrel{-}{A}-\widehat{A}+\stackrel{-}{A}=\stackrel{\sim}{A}.$$

From the well-known algebraic identities^44^ we have

67 $$\:\widetilde A^T{\sigma\:}_A\left(\widehat A-\overset-A\right)=\frac{{\sigma\:}_A}2\left(-\left\|\widetilde A\right\|^2+\left\|A_o\right\|^2-\left\|{\widehat A}_o\right\|^2\right)$$

68 $$\:\widetilde B^T{\sigma\:}_B\left(\widehat B-\overset-B\right)=\frac{{\sigma\:}_B}2\left(-\left\|\widetilde B\right\|^2+\left\|B_o\right\|^2-\left\|{\widehat B}_o\right\|^2\right)$$

69 $$\:e_v\epsilon\:\leq\:\frac12\left(e_v^2+{\epsilon\:}^2\right)\leq\:\frac12\left(e_v^2+{\epsilon\:}_o^2\right).$$

Therefore, after plugging-in (67–69) into (64) we obtain

70 $$\:\begin{array}{c}\dot V=-k_xe_x^2-\left(k_v-\frac12\right)e_v^2-k_ie_i^2+\widetilde A^T{\sigma\:}_A\left(\widehat A-\overset-A\right)+\widetilde B^T{\sigma\:}_B\left(\widehat B-\overset-B\right)+\frac12{\epsilon\:}^2\:\:\:\:\:\:\:\:\:\:\:\:\:\:\:\:\:\:\:\:\:\:\\\:\:\:\:\:\:\leq\:-k_xe_x^2-\left(k_v-\frac12\right)e_v^2-k_ie_i^2-\frac{{\sigma\:}_A}2\left\|\widetilde A\right\|^2-\frac{{\sigma\:}_B}2\left\|\widetilde B\right\|^2+\frac{{\sigma\:}_A}2\left\|A_o\right\|^2+\frac{{\sigma\:}_B}2\left\|B_o\right\|^2+\frac12{\epsilon\:}_o^2\\\:\leq\:-k_{min}\begin{Vmatrix}e_x\\\:e_v\\\:e_{ei}\end{Vmatrix}^2-\frac{{\sigma\:}_A}2\left\|\widetilde A\right\|^2-\frac{{\sigma\:}_B}2\left\|\widetilde B\right\|^2+\frac{{\sigma\:}_A}2\left\|A_o\right\|^2+\frac{{\sigma\:}_B}2\left\|B_o\right\|^2+\frac12{\epsilon\:}_o^2.\:\:\:\:\:\:\:\:\:\:\:\:\:\:\:\:\:\:\:\:\end{array}$$

where $\:{k}_{min}:=\text{min}\left\{{k}_{x},{k}_{v}-\frac{1}{2},{k}_{i}\right\}\:$. The Lyapunov function derivative is therefore negative outside of a compact set $\:E$ defined by

71 $$\:\begin{Vmatrix}e_x\\\:e_v\\\:e_{ei}\end{Vmatrix}^2\leq\:\frac1{k_{min}}\left(\frac{{\sigma\:}_A}2\left\|A_o\right\|^2+\frac{{\sigma\:}_B}2\left\|B_o\right\|^2+\frac12{\epsilon\:}_o^2\right)$$

for any $\:\stackrel{\sim}{A},\:\stackrel{\sim}{B}$ as $\:-\frac{{\sigma\:}_A}2\left\|\widetilde A\right\|^2-\frac{{\sigma\:}_B}2\left\|\widetilde B\right\|^2\leq\:0$. Similarly, the Lyapunov function derivative is negative outside of a compact set

72 $$\:\begin{Vmatrix}\widetilde A\\\:\widetilde B\end{Vmatrix}^2\leq\:\frac2{{\sigma\:}_{min}}\left(\frac{{\sigma\:}_A}2\left\|A_o\right\|^2+\frac{{\sigma\:}_B}2\left\|B_o\right\|^2+\frac12{\epsilon\:}_o^2\right),\:\:{\sigma\:}_{min}=\text{min}\left\{{\sigma\:}_A,{\sigma\:}_B\right\}\:$$

for any $$\:\left[\begin{array}{c}{e}_{x}\\\:{e}_{v}\\\:{e}_{ei}\end{array}\right]$$. According to the well-known extension of the Lyapunov theorem^23^ trajectories of $$\:\left[\begin{array}{c}{e}_{x}\\\:{e}_{v}\\\:{e}_{ei}\end{array}\right]$$ are UUB to the set of attraction $\:E$, which can be narrowed by increasing $\:{k}_{min}$.

The implementation of control defined in (59) requires the errors (42), the reference speed (53) and its derivative, the reference current (58) derived through inversion of the fuzzy model. The parameters of the fuzzy model are adapted according to (62), while the electrical parameters are adapted according to (63). The designer selects: the ranges $\:{x}_{min}$, $\:{x}_{max}$, $\:{i}_{max}$ of the fuzzy model input variables, the controller gains $\:{k}_{x}$, $\:{k}_{v}$, $\:{k}_{i}$ in (53),(58),(59), the adaptation gains $\:{{\Gamma\:}}_{A}$, $\:{{\Gamma\:}}_{B}$, the “robustifying” parameters $\:{\sigma\:}_{A}$, $\:{\sigma\:}_{B}$ and the initial values $\:\stackrel{-}{A}$, $\:\stackrel{-}{B}$ in (62), (63).

**Figure 7:**
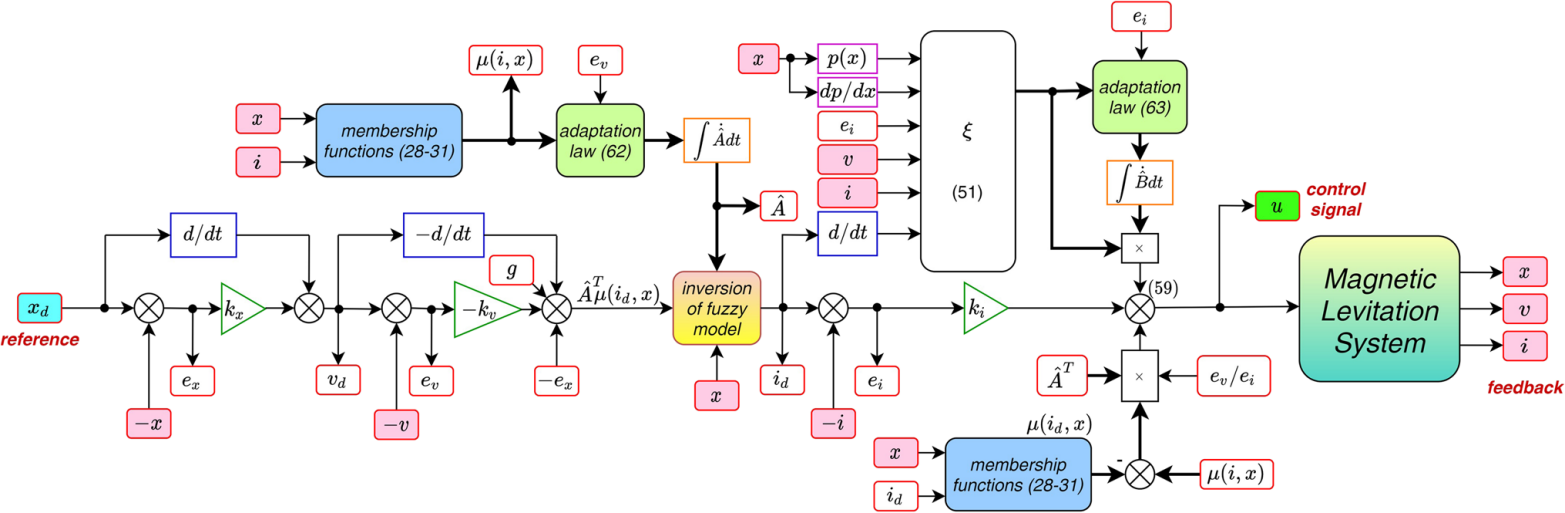
Control algorithm diagram.

The diagram of the proposed control system is presented in Fig. 7.

Finally, let us address some potential numerical issues concerning the proposed control. First, let us notice that division by $\:{e}_{i}$ in (59) does not introduce any singularity for small $\:{e}_{i}$. If $\:i$ and $\:i+{e}_{i}$ activate the same slope of a membership function, say $\:{\mu\:}_{i,{k}_{1}}\left(i\right)$ and $\:\mu\:\left(i,x\right)={\mu\:}_{i,{k}_{1}}\left(i\right)\cdot\:{\mu\:}_{x,{k}_{2}}\left(x\right)$, then $\:\frac{\mu\:\left(i,x\right)-\mu\:\left(i+{e}_{i},x\right)}{{e}_{i}}=\pm\:\frac{1}{{\varDelta\:}_{i}}{\mu\:}_{x,{k}_{2}}\left(x\right)$. Next, the derivative of the desired current $\:{i}_{d}$, which is calculated in (58), is necessary for $\:\xi\:$ in (51). Fortunately, $\:{i}_{d}$ is sufficiently smooth to guarantee that numerical approximation of $\:\frac{d}{dt}{i}_{d}$ by the backward difference is accurate.

## Numerical experiments

Two numerical experiments have been conducted, with two different MLS.

- The first MLS (MLS1) was modelled using the following equations:

73 $$\:\dot{x}=v\:,$$

74 $$\:\dot v\:=g-\frac{L_0x_0}{2M}\frac{i^2}{x^2\:},$$

75 $$\:\left({L}_{\infty\:}+\frac{{L}_{0}{x}_{0}}{x}\right)\frac{di}{dt}=u-iR+{L}_{0}{x}_{0}\frac{vi}{{x}^{2}}\:.$$

So, the MLS1 is of the simplest type, assuming the inductance (4, which implicates the force (5).

- The second device (MLS2) was modelled by the Eq. 

76 $$\:\dot{x}=v,\:$$

77 $$\:\dot v\:=g-\frac KM\frac{i^2}{\left(x+\delta\:\right)\left(x+\delta\:+\delta\:\frac{i^2}{i_{sat}^2}\right)},$$

78 $$\:\left(\frac{2 K}{\left(x+\delta\:+\frac{{i}^{2}}{{i}_{sat}^{2}}\delta\:\right)}-\frac{4 K\delta\:}{{i}_{sat}^{2}}\frac{i}{{\left(x+\delta\:+\frac{{i}^{2}}{{i}_{sat}^{2}}\delta\:\right)}^{2}}\right)\frac{di}{dt}=u-iR+2 K\frac{vi}{{\left(x+\delta\:+\delta\:\frac{{i}^{2}}{{i}_{sat}^{2}}\right)}^{2}}\:,$$

**Figure 8:**
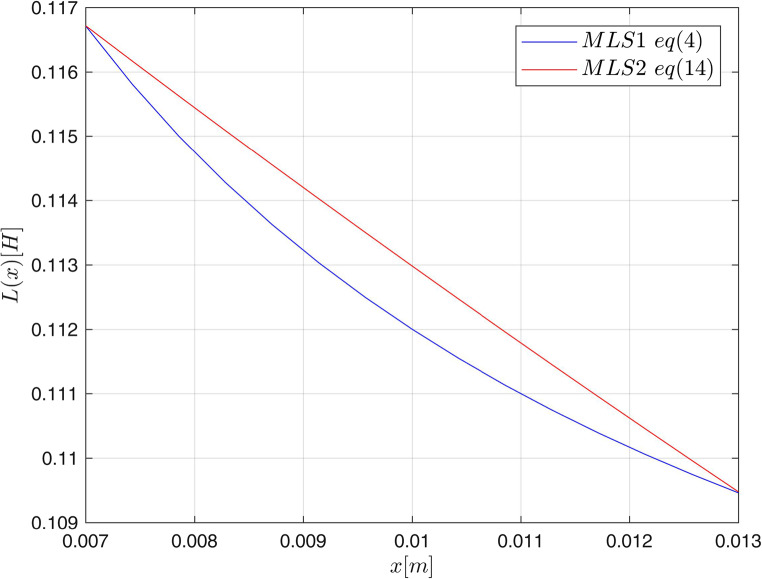
Inductance for MLS1 (4) and MLS2 (14) for $i=1\lbrack A\rbrack$.

**Figure 9:**
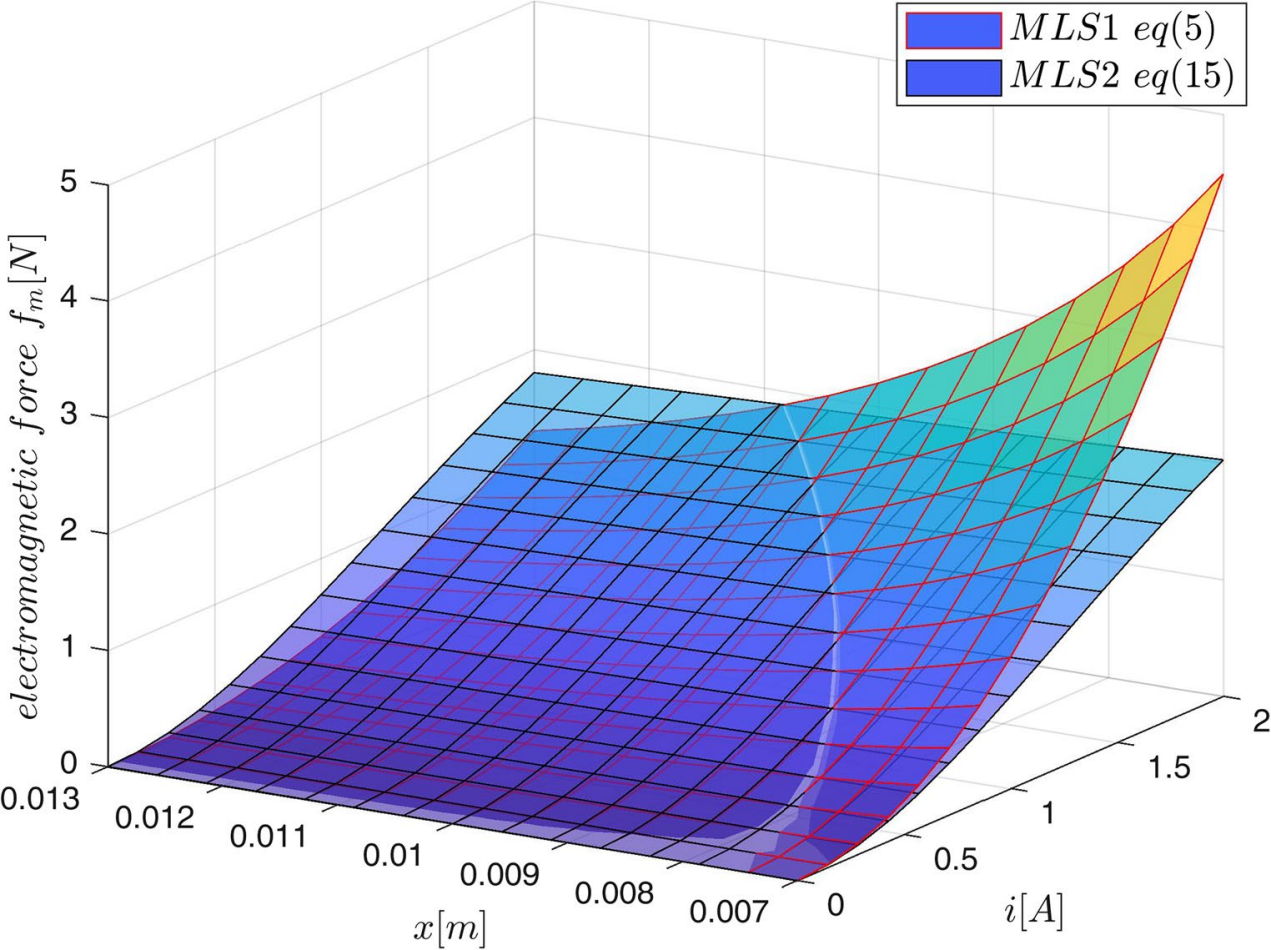
Electromagnetic force for MLS1 (5) and MLS2 (15).

So, in this device, the inductance is described by (14) and the electromagnetic force by (15). Equation (78) is derived directly from $\:\dot{\lambda\:}\left(i,x\right)=u-iR\:$ and $\:\lambda\:\left(i,x\right)=L\left(i,x\right)i$after plugging in formula (14) for $\:L\left(i,x\right)$. The parameters of both MLS are given in Table 3. Inductance $\:L$ and electromagnetic force $\:{f}_{m}$ for both MLS are shown in Figs. 8 and 9.

**Table 3:** Parameters of the modelled MLSs.

$\:M\:\left[kg\right]$	$\:g\:\left[m/{s}^{2}\right]$	$R\:\left[{\Omega\:}\right]$	$\:{L}_{\infty\:}\:\left[H\right]$	$\:{L}_{0}{x}_{0}\:\left[Hm\right]$	$\:K\:\left[\frac{N{m}^{2}}{{A}^{2}}\right]$	$\:\delta\:\:\left[m\right]$	$\:{i}_{sat}\:\left[A\right]$
$\:0.031$	$\:9.81$	$\:3.88$	$\:0.0101$	$1.1\cdot10^{-4}$	0.0053	0.067	$\:2$

The model of the inductance required for controller derivation is $\:L\left(x\right)={L}_{\infty\:}+{L}_{1}p\left(x\right)={L}_{\infty\:}+{L}_{1}{x}^{-1}$, so $\:{L}_{1}$ corresponds to $\:{L}_{0}{x}_{0}$ and $P(x)=x^1$. The model corresponds exactly to the first device - MLS1.

The fuzzy model of the electromagnetic acceleration was trained using $\:40\times\:40$ current and position values uniformly distributed in the rectangle $R=\lbrack0,2\;\rbrack\lbrack A\rbrack\times\lbrack0.007,0.013\rbrack\lbrack m\rbrack$. The acceleration data is obtained from:

79 $$\:f\left(i,x\right)=\frac{{L}_{0}{x}_{0}}{2 M}\frac{{i}^{2}}{{x}^{2}\:},$$

so, corresponds exactly to the first device MLS1.

The accuracy of the fuzzy model, after the initial training, i.e. with parameters $\:\stackrel{-}{A}$, for different number of rules is presented in Table 4.

**Table 4:** Maximum absolute error of the fuzzy model.

*N=m* ^*2*^	9	16	25	49	100	225
${\max\limits_{\mathit(i\mathit(t\mathit)\mathit,x\mathit(t\mathit)\mathit)\mathit\in R}}\vert f(i,x)-y(i,x)\vert\lbrack\frac m{s^2}\rbrack$	7.97	3.14	2.13	0.92	0.43	0.25
$\frac{\max\limits_{\mathit(i\mathit(t\mathit)\mathit,x\mathit(t\mathit)\mathit)\mathit\in R}\vert f(i,x)-y(i,x)\vert}{\max\limits_{\mathit(i\mathit(t\mathit)\mathit,x\mathit(t\mathit)\mathit)\mathit\in R}\vert f(i,x)\vert}100\lbrack\%\rbrack$	5.5	2.17	1.47	0.63	0.30	0.17

The control aim was to track the desired position trajectory

80 $$\:{x}_{d}\left(t\right)={x}_{e}+0.003sin(6\pi\:t-\pi\:),\:{x}_{e}=0.01\left[m\right]$$

with the initial conditions.

81 $$\:x\left(0\right)={x}_{e}\;\;\;v\left(0\right)=0\;\;\;i\left(0\right)=1\;\left[A\right]$$

### Case 1

The device MLS1 was controlled. The fuzzy model was used, with the parameters $\:\stackrel{-}{A}$ obtained from the initial training – so the approximation of the actual acceleration using the fuzzy model was the most accurate. The exact values of parameters $\:B$ were known: $\:\stackrel{-}{B}=B$. The applied control parameters were:

$$\:k_x=100,\:k_v=10,\:k_i=10,\:{\Gamma\:}_\text{A}=10^3\cdot1_{NxN},\:{\sigma\:}_A=10^{-2},\:{\Gamma\:}_\text{B}=diag\left(1,\:10^2,10^{-5}\right),\:{\sigma\:}_B=10^{-2}.$$

**Figure 10:**
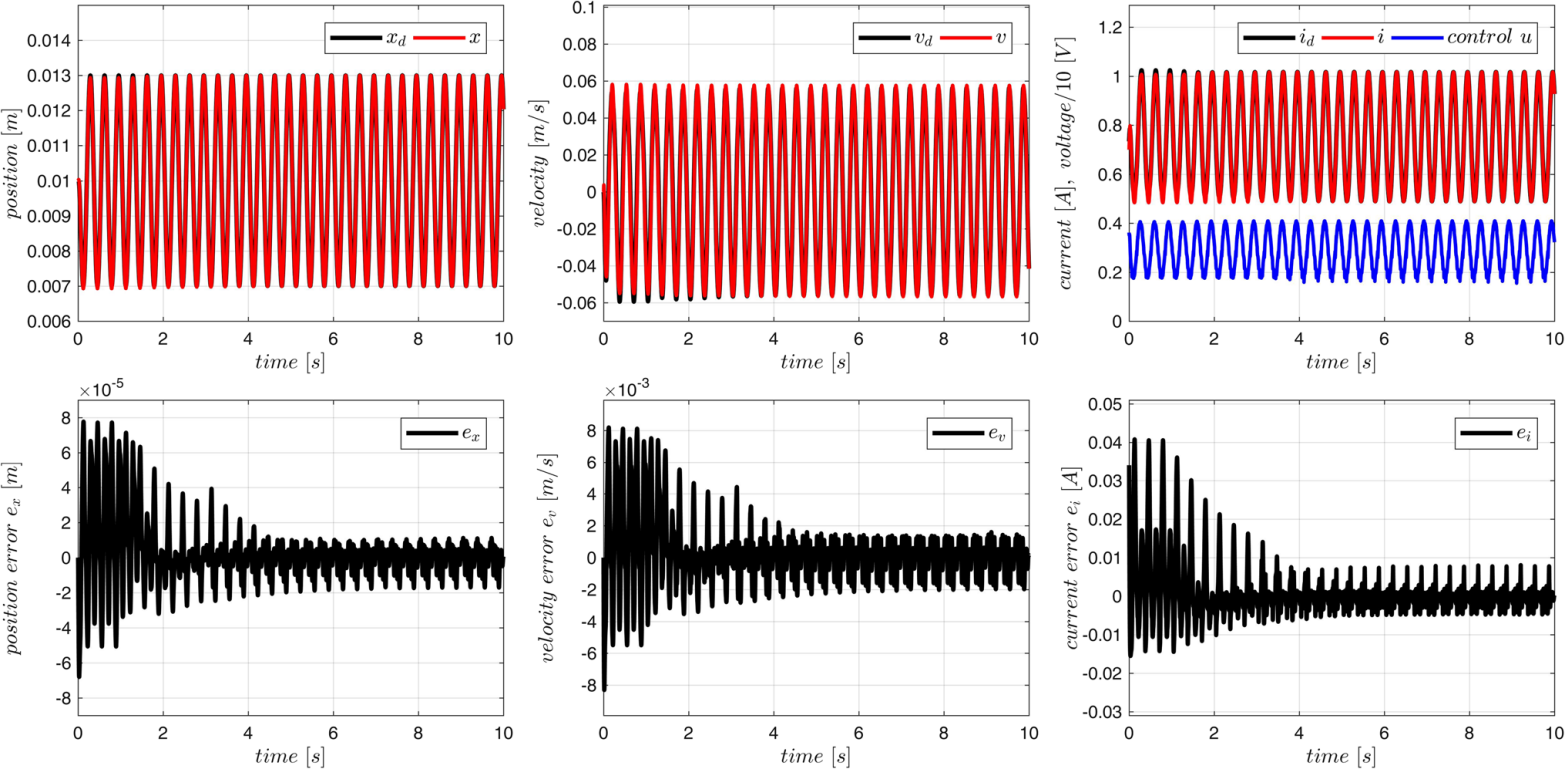
Case 1. Tracking errors for number of rules $\:N={5}^{2}$. Adaptation started at $\:{t}_{a}=1\:\left[s\right]$.

**Figure 11:**
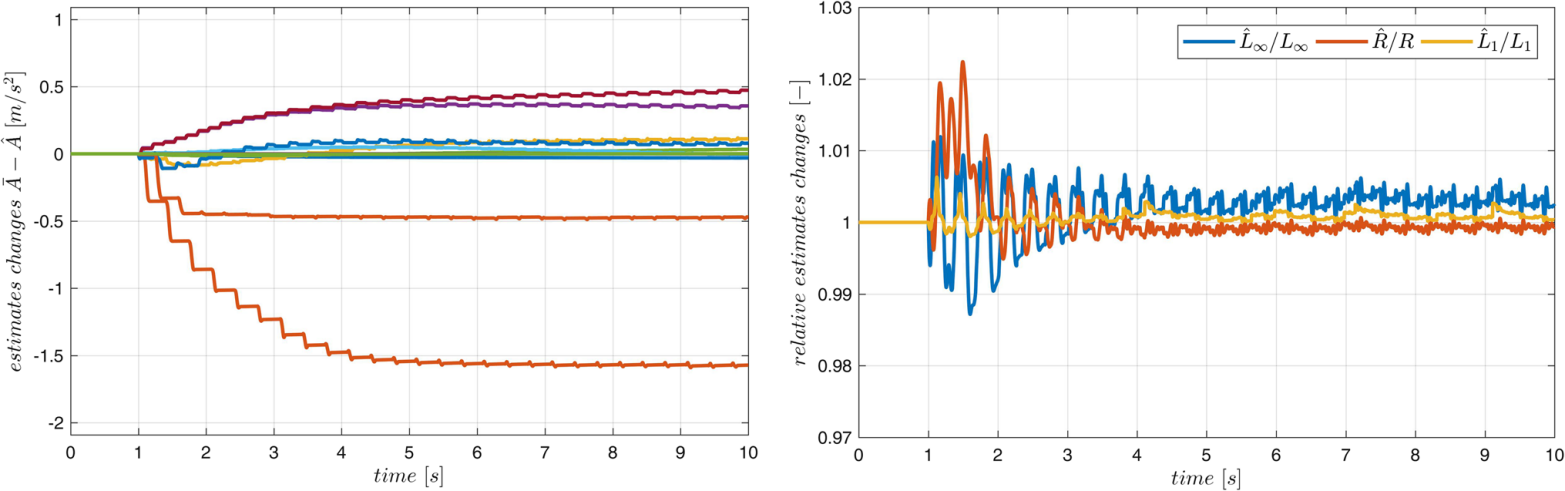
Case 1. Parameters $\:\widehat{A}$ and $\:\widehat{B}$ during the on-line adaptation.

Typical plots obtained from the system operation are presented in Figs. 10 and 11. On-line adaptation was started at $\:{t}_{a}=1\:\left[s\right]$. A few seconds later the tracking errors decrease below acceptable limits (Fig. 10). Adaptive parameters $\:\widehat{A}$ and $\:\widehat{B}$ remain bounded, as it is presented in Fig. 11. Adaptive parameters values change slightly, as the starting values were almost exact. Especially, parameters of the ‘electrical’ Eq. (19) are almost constant – the adaptation of $\widehat B$ can be switched off if initial values $\:\stackrel{-}{B}$ are accurate.

The control $u$ and all the state variables are easily kept inside available constraints imposed by the real plant.

The system behaviour was examined for different number of rules used in the fuzzy model of acceleration. Results are summarized in Table 5. Both position tracking errors (before and after on-line adaptation) decrease with the increasing number of rules, and in any case the error after the adaptation is smaller than the error before.

**Table 5:** Position tracking error before and after the on-line adaptation for different number of rules $\:N$ of the fuzzy model.

$N={m}^{2}$	$\:9$	$\:16$	$\:25$	$\:49$	$\:100$	225
$\:\underset{0.1<t<1}{\text{max}}\left|{e}_{x}\left(t\right)\right|\:\left[\mu\:m\right]\:$	$\:336$	$\:170$	$\:78$	$\:33$	13	$\:6$
$\:\underset{t>10}{\text{max}}\left|{e}_{x}\left(t\right)\right|\:\left[\mu\:m\right]\:$	$\:58$	27	$\:17$	$\:9$	$\:7$	$\:4$

### Case 2

**Figure 12:**
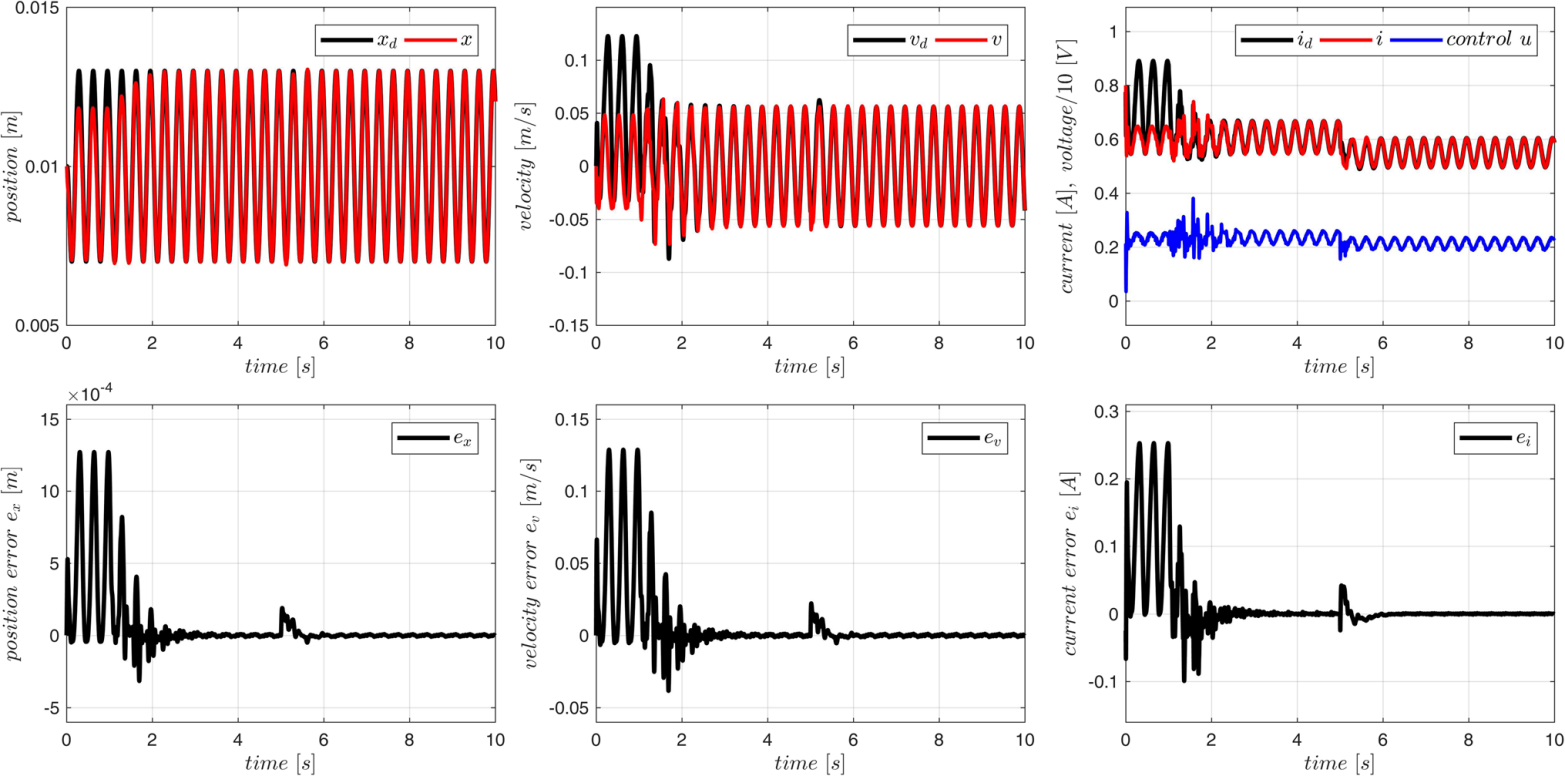
Case 2. Tracking errors for number of rules $\:N={5}^{2}$. Adaptation started at $\:{t}_{a}=1\:\left[s\right]$. The mass was changed at $\:{t}_{m}=5\:\left[s\right]$.

**Figure 13:**
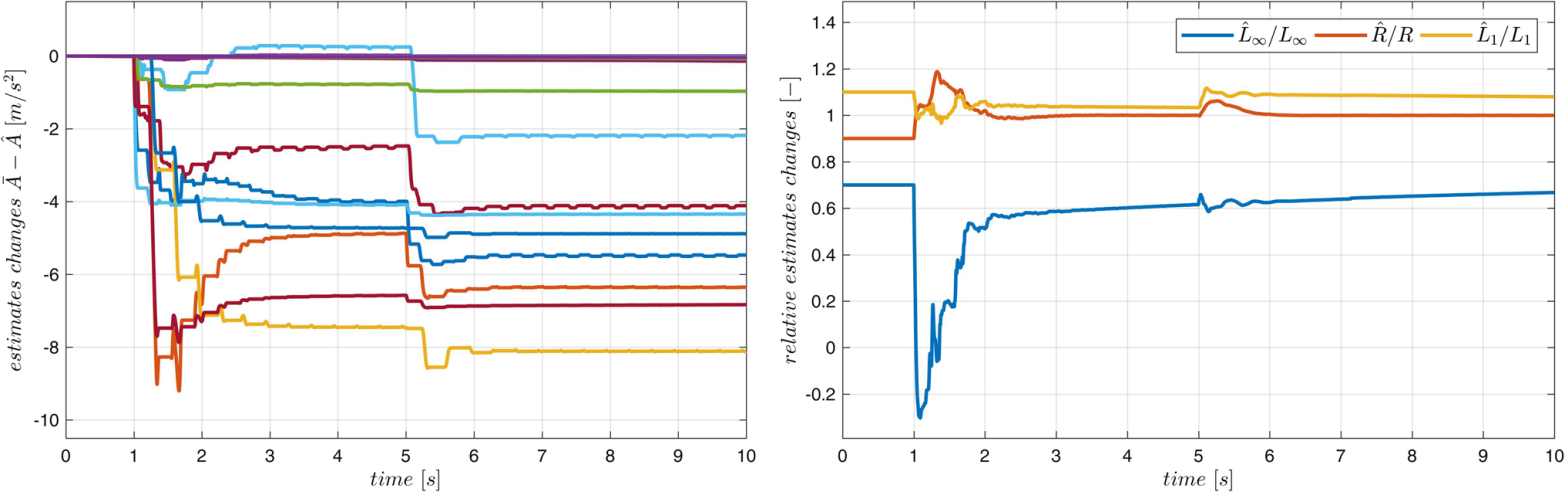
Case 2. Parameters $\:\widehat{A}$ and $\:\widehat{B}$ during the on-line adaptation.

**Figure 14:**
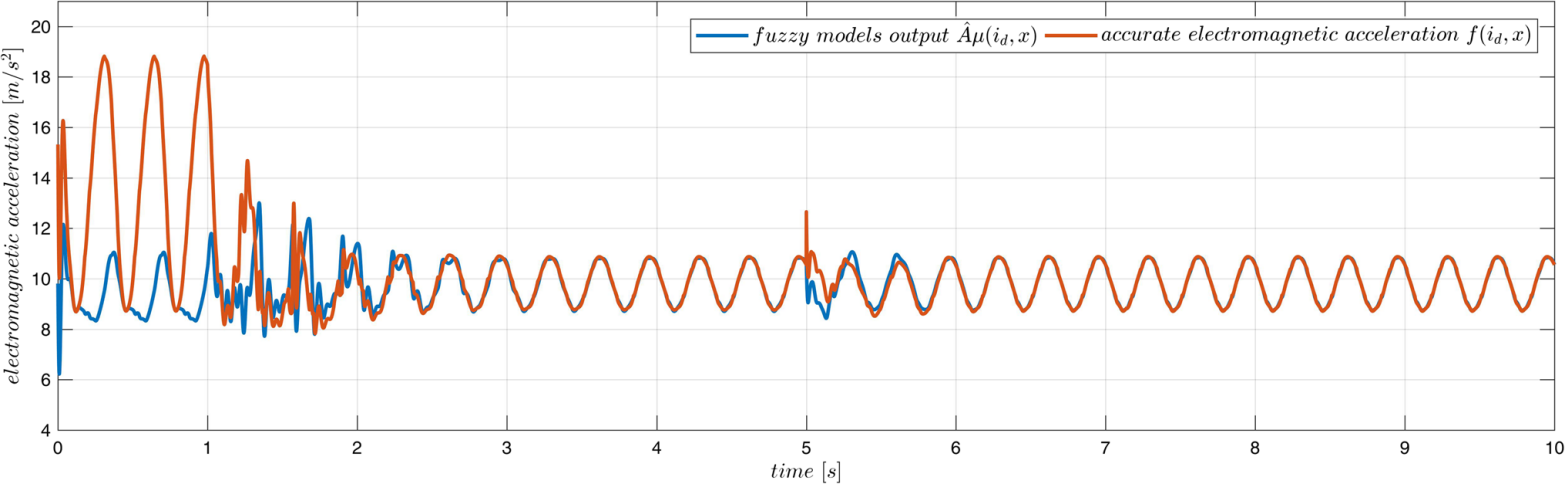
Case 2. Exact electromagnetic acceleration and the output of the fuzzy model ($\:N={5}^{2})$. Adaptation started at $\:{t}_{a}=1\:\left[s\right]$. The mass was changed at $\:{t}_{m}=5\:\left[s\right]$.

The device MLS2 was controlled. Equations (76–78) describing this device contain several parameters ($\:{l}_{fe},{i}_{sat}$), which cannot be easily derived from experiments, measurements or the manufacturer data. Therefore, it is difficult or even impossible to propose any accurate fuzzy model of the acceleration in MLS2. We use the same fuzzy model of acceleration and the same model of the coil inductance as in Case 1 to control MLS2. Additionally, to increase the degree of inconsistency of the model with the MLS, we reduce the initial parameters $\overline A$ by 10% and we accept inaccurate initial values of parameters $\:{B}^{T}=[{L}_{\infty\:},R,{L}_{1}]$: ${\overline L}_\infty=0.7L_\infty$, $\:\stackrel{-}{R}=0.9R$, $\overline{L_1}=1.1L_1=1.1L_0x_0$. The applied control parameters were: $\:{k}_{x}=100$, $\:{k}_{v}=10$, $\:{k}_{i}=10$, $\Gamma_A=3\cdot10^3\cdot1_NxN$, $\:{\sigma\:}_{A}={10}^{-2}$, $\:{{\Gamma\:}}_{\text{B}}=diag\left(1,\:{10}^{2},{10}^{-5}\right)$, $\:{\sigma\:}_{B}=1{0}^{-2}$. Typical plots obtained from the system operation are presented in Figs. 12, 13 and 14. On-line adaptation was started at $\:{t}_{a}=1\:\left[s\right]$. At $\:{t}_{m}=5\:\left[s\right]$ the mass of the moving ball was rapidly decreased by 20%. A few seconds after starting the adaptation, but before changing the mass of the ball, the tracking error was smaller than 20$\:\mu\:m$. Five seconds after changing the mass of the ball the adaptive control decreases the tracking errors below the same limits (Fig. 12) as in Case 1, were the more accurate model was used from the very beginning. As in Case 1, all adaptive parameters (Fig. 13), control, and state variables (Fig. 12) remain bounded during the system operation. The evident improvement of the fuzzy model accuracy, due to the on-line adaptation, is presented in Fig. 14.

Let us notice that during this experiment not only the starting fuzzy model of the acceleration was inaccurate. Also the model of the inductance accepted for the control derivation $\:{L\left(x\right)=L}_{\infty\:}+{L}_{1}{x}^{-1}$ was far from the actual one used in (78). Despite all this uncertainty, the on-line adaptation was powerful enough to ensure proper operation of the closed loop system.

## Real plant experiments

**Figure 15:**
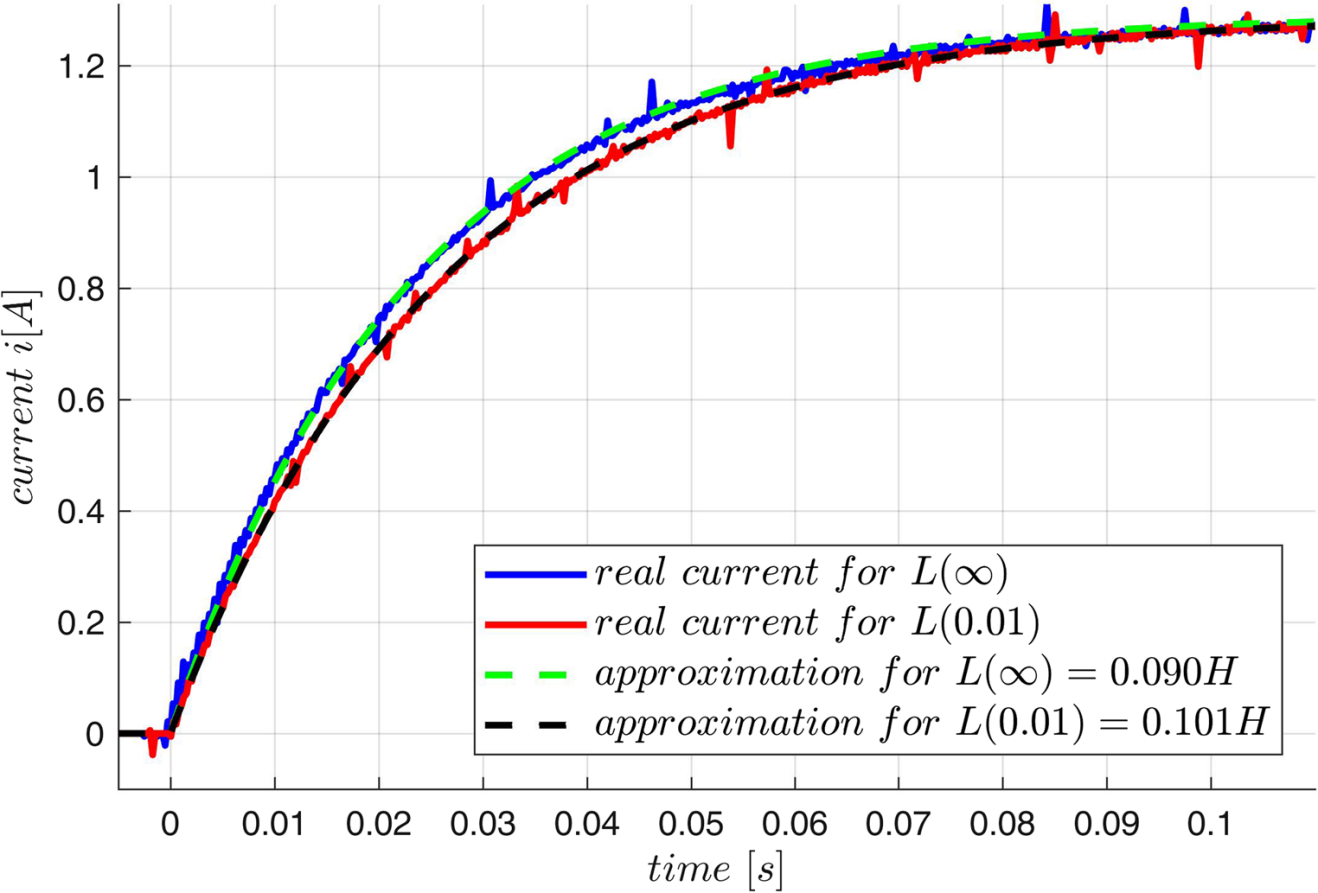
Current plots during identification experiments.

**Figure 16:**
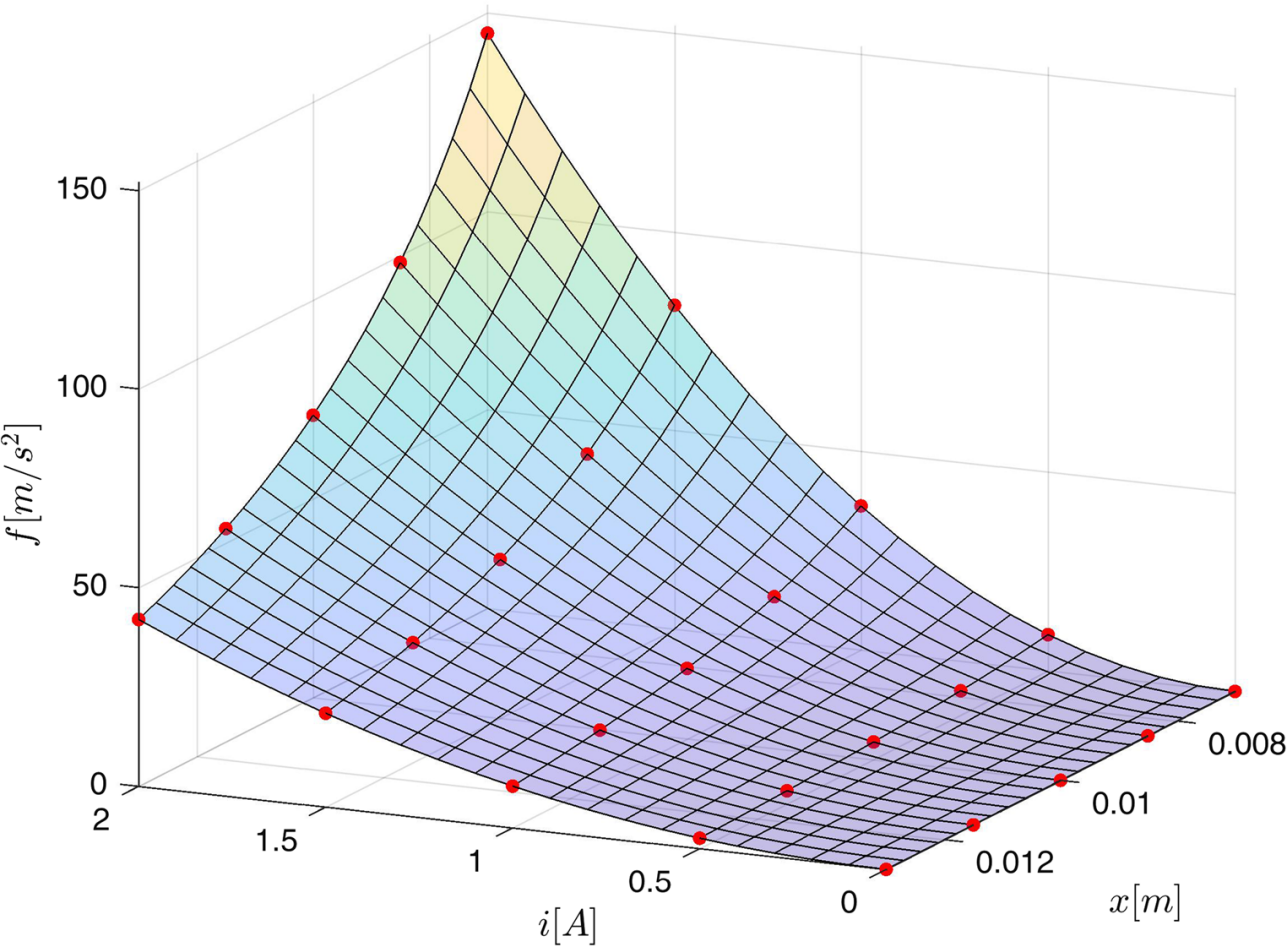
Initial values of the 25-rule fuzzy model parameters (red dots) on the background of the surface$f(i,x)=\frac{L_0x_0}{2M}\frac{i^2}{x^2}.$

The proposed control approach was tested with a real MLS produced by INTECO and presented in Fig. 1. The mass of a moving ball was $\:M=0.031 kg$. First, a simple identification of the electrical equation was performed. Two step responses ($\:u\left(t\right)=5\cdot\:1\left(t\right)$) of the current were recorded: during the first one the ball was moved away from the plant, during the second it was positioned ($\:v=0$) at $\:x=0.01 m$. The plots are presented in Fig. 15. At the steady-state the current was $\:{i}_{set}=1.29 A$, hence the resistance was estimated as $\:\stackrel{-}{R}=5/{i}_{set}=3.88{\Omega\:}$. As the theoretical current for a constant inductance $\:L$ is: $\:i\left(t\right)={i}_{set}\left[1-\text{exp}\left(\frac{tR}{L}\right)\right]$, this very rough model of the inductance allows to estimate by the curve fitting $\:{L}_{\infty\:}=0.090\:\left[H\right],\:\:L\left(0.01\right)=0.101\:\left[H\right]$. Assuming the simplest model of $\:L\left(x\right)={L}_{\infty\:}+\frac{{L}_{0}{x}_{0}}{x}$ we get $\:L_1=L_0x_0=1.1\:\cdot\:10^{-4}\:\:\left[Hm\right],\:\:p\left(x\right)=1/x$. This data are enough to propose a very inaccurate model of the electromagnetic acceleration $\:f(i,x)=\frac{{L}_{0}{x}_{0}}{2 M}\frac{{i}^{2}}{{x}^{2}}$, and this was used to construct the fuzzy model $\:f(i,x)\approx\:{A}^{T}\mu\:\left(i,x\right)$ with $\:N=25$ rules with initial parameters $\:\stackrel{-}{A}=\left[{\stackrel{-}{a}}_{j}\right]$, where $\:{\stackrel{-}{a}}_{j}=\frac{{L}_{0}{x}_{0}}{2 M}\frac{{i}^{2}}{{x}^{2}}$ for such $\:i,x$ that $\:{\mu\:}_{j}(i,x)=1$. The obtained surface and the parameters located at the appropriate $\:i,x$ are shown in Fig. 16.

The control was implemented using a DSP board *dSPACE* DS1104 with the sampling time $\:100\mu\:s$. The necessary derivatives of the desired current and the ball velocity were obtained from simple differential filters with the transfer function $G_{fid}(s)=\frac s{0.001s+1}$ and $\:{G}_{{f}_{x}}\left(s\right)=\frac{s}{0.01s+1}$ appropriately.

The applied control parameters were $$\:{k}_{x}=100$$, $$\:{k}_{v}=10$$, $$\:{k}_{i}=50,$$$$\:{\Gamma\:}_\text{A}=10^3\cdot\:1_{NxN}$$, $$\:{\sigma\:}_{A}={10}^{-3}$$, $$\:{{\Gamma\:}}_{\text{B}}=diag\left(1,\:{10}^{2},{10}^{-5}\right)$$, $$\:{\sigma\:}_{B}=1$$, $$\:{\Gamma\:}_\text{B}=10^{-9}\cdot\:1_{NxN}$$. The reference trajectory (80) was used.

**Figure 17:**
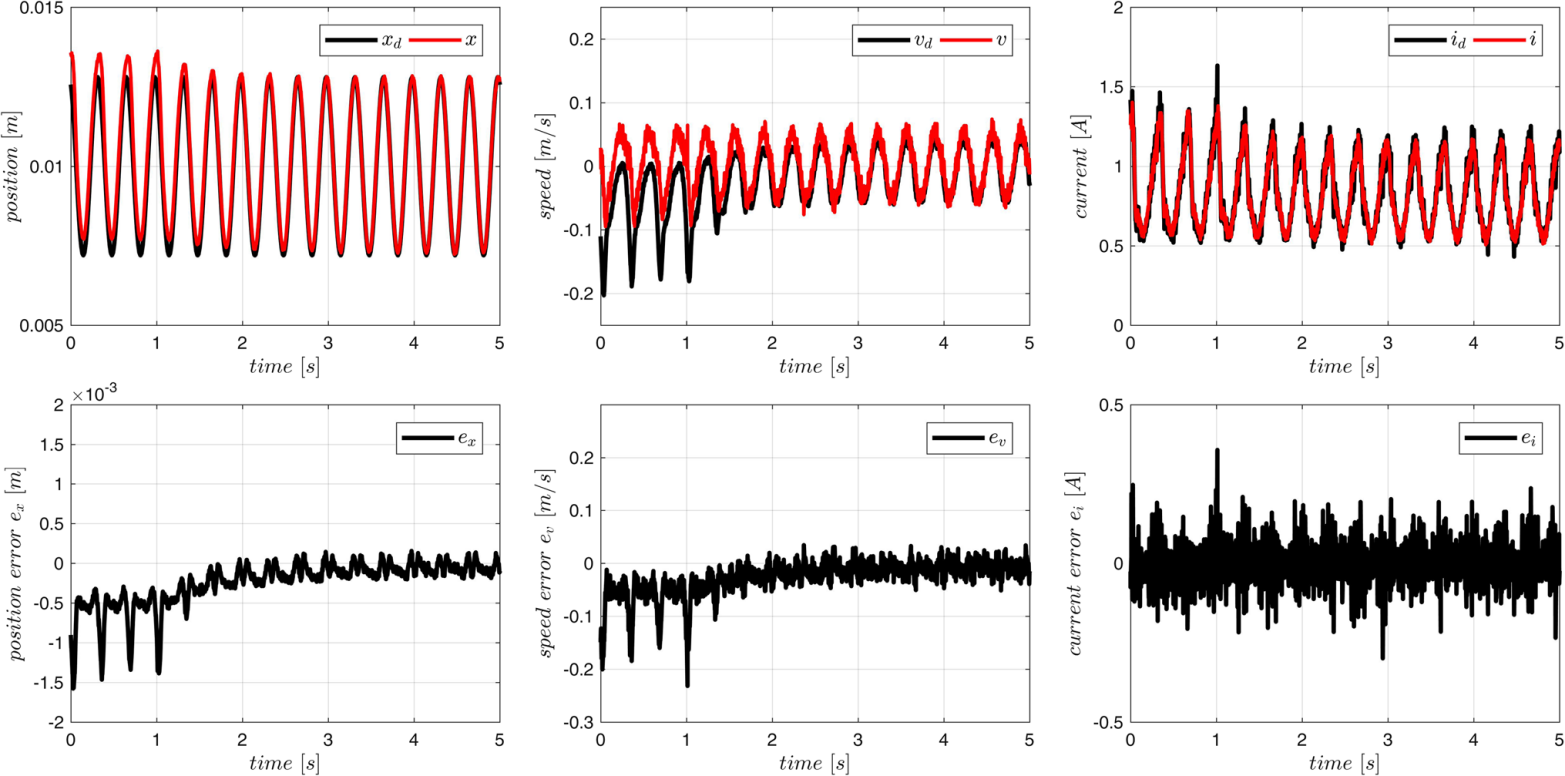
Tracking performance during experiments with the real plant.

**Figure 18:**
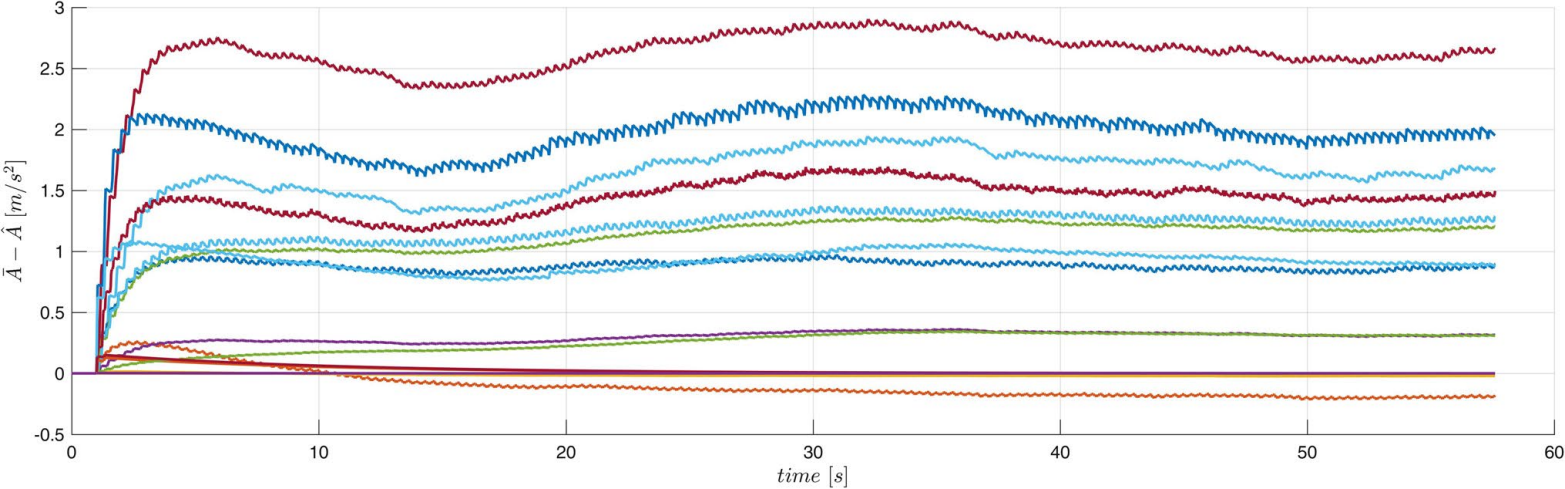
Changes of the parameters of the fuzzy model during adaptation.

The tracking ability of the proposed controller is presented in Fig. 17. All state variables track the required trajectories with the sufficient accuracy. In position tracking, the error is limited by the measurement accuracy rather than the control capabilities. Also during experiments with the real plant all adaptive parameters (Fig. 18), state variables and control remain inside the constraints imposed by physical properties of the hardware.

The controller proposed here has been compared with several control algorithms described in the literature. We selected several state-of-the-art algorithms that do not require performing numerous identification experiments, similarly to the approach presented here. Therefore, algorithms based on genetic optimization, requiring multiple runs, and those requiring prior offline data collection (supported by neural networks, fuzzy or neuro-fuzzy systems) were rejected. Finally, four approaches have been compared:

- PID – a standard PID controller based on position error, proposed by the manufacturer of the MLS,
- LQR -linear quadratic regulator based on the linearized model (73–75) as described in^45^.
- SMC -sliding mode controller as described in^46^,
- AB – the adaptive controller based on the fuzzy inversion presented here with parameters selected as described above in this section.

As it was impossible to obtain stable operation of all tested controllers for the reference (80) because of the wide range of position changes required by the trajectory (80) we have decided to select the reference $\:{x}_{d}\left(t\right)$ as the output of the filter described by the transfer function $\:{G}_{ref}\left(s\right)=1/{\left(0.1s+1\right)}^{3}$ excited by the periodical signal

82 $$\:{x}_{pf}\left(t\right)=\left\{\begin{array}{c}{x}_{0}-0.001\:for\:k\frac{\pi\:}{2}\le\:t<\left(k+1\right)\frac{\pi\:}{2}\:if\:k=0,\:2,\:4,\dots\:\\\:{x}_{0}+0.001\:for\:k\frac{\pi\:}{2}\le\:t<\left(k+1\right)\frac{\pi\:}{2}\:if\:k=1,\:2,\:5,\dots\:\end{array}\right.,\:\:\:{x}_{0}=0.01\:\left[m\right].$$

**Figure 19:**
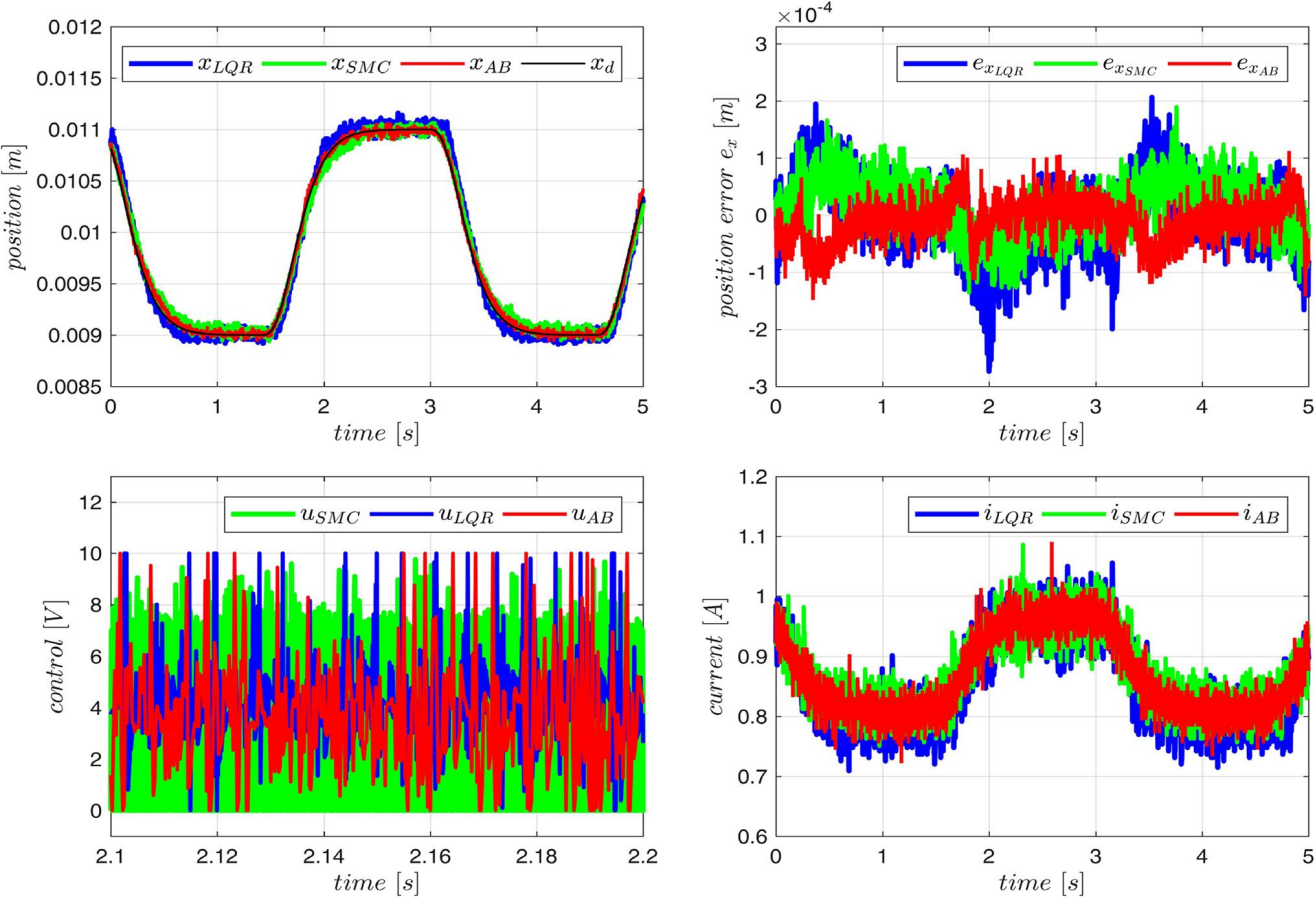
Comparison of three control algorithms.

This reference is presented in Fig. 19. The experience with the tunning of the tested controllers can be summarized with the following conclusions:

- PID – Stable operation was possible only for a constat reference, any change of the reference requires new parameter tunning, hence, this controller was excluded from further experiments.
- LQR – It was necessary to add an integral action to eliminate the steady-state error, finally the applied control was $\:u=-{k}_{1}\left(x-{x}_{e}\right)-{k}_{2}v-{k}_{3}\left(i-{i}_{e}\right)-{k}_{4}\int\:\left(x-{x}_{e}\right)dt+{u}_{e}$, where the index $\:{*}_{e}$ represents steady-state conditions where the control $\:{u}_{e}$ generates the current $\:{i}_{e}$ producing the electromagnetic force compensating for the gravity at the position $\:{x}_{e}$. Many attempts to select weighting factors in the quality index and manual tunning of $\:{u}_{e}$ and $\:{i}_{e}$ were necessary to obtain correct operation of the closed-loop system with the assumed reference.
- SMC - Similarly to LQR, a component related to the error integral was introduced, which was used in the equation of the switching hyperplane.

All tested controllers were tuned carefully to minimize the tracking error preserving the similar energy consumption measured by the index $E=\int_0^5Ri^2(t)dt$ . The tracking abilities were compared by indices $\:RMS{E}_{ex}=\sqrt{\frac{\sum\:_{{t}_{k}=1}^{50000}{e}_{x}^{2}\left({t}_{k}\right)}{50000}}$ and $\:{e}_{MAX}=\frac{\underset{0\le\:t\le\:5}{\text{max}}\left|{e}_{x}\left(t\right)\right|}{{x}_{e}}100\%$ using 50,000 samples with the sampling time $\:100\mu\:s$. The control quality was compared using the index$RMSE_u=\sqrt{\frac{\sum_{t_k=1}^{50000}u^2(t_k)}{50000}}$ . The comparison of the tested systems is presented in Table 6 and the exemplary plots are given in Fig. 19. The tracking error $\:RMS{E}_{ex}$ for the presented controller equals 57% of the same index for LQR and 75% for SMC. Similar relation is observed for $\:{e}_{MAX}$. What is more, adaptive controller achives the best tracking with the lowest value of $\:RMS{E}_{u}$. The amplitude of current oscillations is also the smallest for the adaptive controller.

**Table 6:** Performance of three control systems.

	$\:LQR$	$\:SMC$	$\:AB$
$\:RMS{E}_{ex}\left[m\right]$	$\:5.8\cdot\:{10}^{-5}$	$\:4.4\cdot\:{10}^{-5}$	$\:3.3\cdot\:{10}^{-5}$
$\:{e}_{MAX}\:\left[\%\right]$	$\:2.36$	$\:2.04$	$\:1.56$
$\:RMS{E}_{u}\left[V\right]$	$\:4.2$0	$\:4.51$	$\:4.13$
$\:E\:\left[W\right]$	$\:15.7$	$\:15.8$	$\:15.8$

## Discussion

The developed controller is based on a special fuzzy model of the electromagnetic acceleration which enables fast execution time and fast and efficient accurate inversion. Previously reported applications of fuzzy theory to MLS control concentrate on fuzzy improvement of the classical (PID or state-feedback) controller parameters while changing the set-point. Here the fuzzy model is connected with the non-standard adaptive control scheme. Fuzzy model parameters are tunned on-line as well as electrical circuit parameters. Therefore it was possible to start with a verry inaccurate model and to obtain perfect tracking, what was demonstrated in numerical experiments and practical implementation.

The UUB stability is proved, what is sufficient for practical applications. It means that the tracking errors are convergent to a certain compact set. The inequalities (71) and (72) are rather conservative conditions defining this set. The point is, that increasing design parameters $\:{k}_{x}$, $\:{k}_{v}$, $\:{k}_{i}$ we can decrease the diameter of the limit set for the errors almost arbitrarily. At the same time the “leakage” parameters $\:{\sigma\:}_{A}$, $\:{\sigma\:}_{B}$ can increase the amplitude of the quasi-steady-state tracking error, so the designer has to compromise between those two factors. For the practical implementation presented here it was quite easy to tune the parameters such that the further decrease of the tracking error is limited by the accuracy of measurement equipment used.

The plant is in a pure-feedback form, so the control based on a strict-feedback system form, like backstepping, is not applicable. The proposed approach is based on a specially tailored adaptive control scheme. Because of adaptive laws (62) and (63) it is partially similar to regressor-based adaptive control. Some references^47^ claim that, for instance, a conventional adaptive control scheme for manipulators requires computation of the regressor matrix, persistent excitation condition of the reference input signal due to convergence of the parameter vector, and slow behavior of system dynamic. It was not observed in the problem discussed here. Of course, the controller requires measurement of state variables, but not problems with persistent excitation or slow behavior were observed. The regressor-free adaptive controllers proposed for robotic arms are based on robust adaptive control approach. It possesses its own limitations and, although it is tempting to avoid measurements, we must leave the comparison of these different control strategies to a separate study.

The numerical complexity of the proposed controller is higher than of standard linear controllers. The computational load is similar to LQR-based controllers or nonlinear controllers. Despite this, it was possible and convenient to implement the controller with the sampling-time typical for modern DSP boards - $\:100\mu\:s$. This value was even too short in comparison with system time constants (for instance the current time-constant is $\:\sim0.2\:\left[s\right]$) and it was possible to demonstrate the it is possible to implement the proposed algorithm in such a short time. The sampling time can be such short due to the structure of the applied fuzzy model, even with a large number of rules. The particular selection of design parameters like weighting matrices and control gains does not influence the computational load of the algorithm and so, the necessary execution time.

Obviously, the measurement noise is noticeable in practical implementation. It depends on the quality of the equipment used and is unavoidable in any real system. The space for improvement of the control quality (while remaining in the proposed control scheme) lays within more sophisticated parameter tunning (maybe using some optimization methods. Higher quality of the measurement equipment (optical position sensor, current sensor) and A/D converters is a crucial factor which can improve practical implementation much.

## Conclusions

According to the aim of the paper we have developed a novel adaptive tracking controller for MLSs based on a special adaptive control scheme incorporating fuzzy model of electromagnetic acceleration enabling fast and accurate fuzzy inversion.

The performed tests and experiments demonstrate that the proposed control technique is robust against discretization and unknown MLS model parameters, provides high tracking accuracy, is easily implementable, and simple to tune.

The control was performed using a fuzzy model of the nonlinear acceleration generated by the electromagnet and an accurate technique of the fuzzy model inversion. The proposed simple fuzzy model allowed for its fast inversion in order to calculate the current needed to generate the desired acceleration of the levitating sphere, which allowed for real-time operation even for a system with such fast dynamics. The tracking accuracy is correlated with the accuracy of the fuzzy model, which increases with the number of rules. A more accurate model allows for more precise determination of the current generating the desired acceleration. Due to the structure of the fuzzy model, even a large number of rules does not disqualify the usability of the solution presented in the paper and does not extend the required sampling time.

Simulation studies and experiments in a real system have shown that the adaptive mechanism of the control algorithm allows the control system to be started without precise knowledge of the system parameters and the shape of the modelled electromagnet force. However, more accurate determination of the initial estimates of the system parameters and the fuzzy acceleration model parameters reduces the control effort and overshoot in transient states. It also allows to limit the fluctuations of the adaptive parameters. Although the MLS is subject to natural constraints on the state and input variables (for position, speed, current, and voltage), which are not explicitly considered in the controller derivation, it was easy to tune the controller parameters so that these constraints were never violated.

Let us also notice that the described application in the control of a typical laboratory MLS system confirms that even complex and advanced control algorithms can be successfully demonstrated and tested using simple equipment available to engineering students worldwide.

The proposed approach constitutes the interesting alternative for standard, linear controllers, especially in the case of large range of motion, complex magnetic circuit, unknown parameters. Practical applications of the proposed approach concern all magnetic suspension problems starting from a standard electromagnetic MAGLEV suspension till active reduction of oscillations of a vehicle.

Of course the proposed approach possesses some limitations. It was formulated and derived for an electromagnetic suspension with one coil. The reference trajectory must be smooth enough. The controller requires measurement of state variables and the quality of these signals has a significant impact on the control quality. The control and state constraints were not taken into account directly during derivation of the controller, although it was not difficult to observe actual constraints by the proper parameter tunning. Those limitations indicate directions of further research.

The proposed approach is based on a concept of stability-based control. An interesting alternative is so-called prescribed performance control (PPC), where the tracking error must satisfy predetermined constraints. Numerous works propose different approaches to PPC. Recently, PPC with fault-tolerant control was described in^48^. The controller is based on fuzzy approximation of nonlinearities and backstepping. The result is restricted to strict-feedback systems (which MLS is not), and the inversion of the fuzzy model is not necessary. The approach based on the fuzzy inversion proposed here can be a good starting point to remove the assumption of strict-feedback form. This indicates a possible direction for future work.

The proposed technique can be successfully used to control more complex systems using the magnetic levitation phenomenon. For example, the magnetic circuit may be more complex, the inductance model of the coil may be more complicated, or the action of several electromagnets on the body may be considered. The application of observers together with the proposed adaptive approach should be investigated to reduce impact of measurement quality. The similar result can be obtained by using nonlinear robust control techniques which should be compared with the proposed approach.

## Data Availability

The datasets generated during and analysed during the current study are available from the corresponding author on reasonable request.
